# Developmental-envelope screening and genome-annotation audit prioritize chemical authenticity candidates in commercial *Rubus chingii* fruit

**DOI:** 10.1016/j.fochx.2026.104635

**Published:** 2026-10-16

**Authors:** Wenkai Duan, Zhenxun Wei, Wenli Gao, Lingyao Peng

**Affiliations:** a Hangzhou Polytechnic University, Hangzhou 310018, China; b Hangzhou TCM Hospital Affiliated to Zhejiang Chinese Medical University, Hangzhou 310007, China

**Keywords:** *Rubus chingii* fruit, Food chemistry, Maturity, Flavour volatiles, Flavonoids, Genome annotation

## Abstract

Commercial dried*Rubus chingii* (Chinese raspberry) fruit is traded by producing region, but unknown harvest maturity can generate compositional ranges that resemble provenance differences. We characterised nine producing-location groups using HS-SPME-GC–MS, LC-MS/MS, amino-acid analysis, electronic-nose sensing and report-derived electronic-tongue outputs, and then applied two sequential evidence gates: developmental-envelope screening of chemical candidates and species-specific annotation auditing of chemistry-linked gene families. The resource contained 124 volatile rows, 318 putatively annotated LC-MS/MS rows, 17 quantified amino acids and 14 electronic-nose channels. In an independent public dataset of 24 fresh-fruit samples (six per stage), 20 of 21 metabolites were stage-resolved and all 11 cross-dataset overlaps were stage-sensitive. The observed four-stage envelope covered the complete nine-location range for eight overlaps. Gallic acid and ellagic acid retained positive range excess in 8/9 and 9/9 leave-one-location-out analyses and exceeded the 95th percentile in all three moderate drying scenarios, defining Tier 1 validation candidates. Kaempferol 3-O-glucoside was retained in 9/9 analyses but exceeded that scenario threshold in 1/3 settings, defining Tier 2. Whole-proteome screening identified 178 PF00201 UGT candidates, recovered all 172 previously catalogued RchUGTs and added six profile-defined candidates; 32 TPS candidates had motif support. Comparative analysis in *R. idaeus* and *R. occidentalis* independently supported re-curation of the LG03.2235 boundary, whereas LG02.1599 remained consistent with a single homologous model. This two-gate workflow converts missing maturity metadata and incomplete genome labels into explicit validation priorities without presenting the candidates as established origin markers, causal regulators or enzyme assignments.

## 1 Introduction

*Rubus chingii* Hu is a traditional medicinal and edible resource in China whose aggregate fruits contain aroma volatiles, amino acids and flavonoids that contribute to their compositional and sensory profile (Chen et al., 2021; Li et al., 2021; Sheng et al., 2020; Xiong et al., 2024; Yu et al., 2019). Zhejiang material is traded as a representative authentic (*daodi, 道地) medicinal product within the Zhe Ba Wei (浙八味) group (*Zhao et al., 2012*) — the dried fruit corresponds to the official drug Rubi Fructus (覆盆子, fupenzi) — and commercial dried fruits are traded* from multiple producing regions in eastern China, yet harvest maturity is often not traceable after drying and distribution. Because fruit development and processing can alter berry phytochemicals and their measured bioavailability (Teegarden et al., 2019; Toydemir et al., 2022), an apparent between-region difference may reflect maturity, drying or storage rather than provenance. The primary food-authenticity question is therefore: among commercial dried fruits of unknown harvest maturity, which chemical differences, if any, exceed the variation attributable to fruit development and consequently merit independent validation as candidate authenticity markers?

Answering this question requires more than demonstrating separation among the available lots. Single compounds can vary with maturity, processing and storage, while electronic noses and tongues provide holistic fingerprints whose interpretation depends on instrument-quality information, chemical anchoring and correct separation of technical from biological replication (Ciosek & Wróblewski, 2007; Rock et al., 2008). High-dimensional targeted and non-targeted workflows can likewise produce optimistic or non-transferable discrimination when validation does not reflect the intended commercial use (Cavanna et al., 2018; García-Pérez et al., 2024; McGrath et al., 2018; Zhong et al., 2022). A defensible workflow must therefore describe the observable commercial-fruit chemistry, quantify how much of each apparent location range is covered by developmental variation and reserve marker status for subsequent stage-controlled validation.

GC–MS, chromatographic profiling, amino-acid chemometrics and electronic sensing have all contributed to fruit characterisation and authenticity research (Haider et al., 2024; Wistaff et al., 2021; Xu et al., 2020; Zhao et al., 2023). However, commercial-lot differences and validated provenance markers are not equivalent evidential endpoints. Here, maturity was treated as a prespecified attribution gate: a feature could be prioritised only when its observed nine-location range exceeded the developmental range measured in an independent four-stage dataset, with scenario analysis used to expose sensitivity to unknown stage mixtures and drying variability. This design tests marker candidacy under missing harvest-stage information; it does not reconstruct the harvest history of individual lots or establish a transferable origin classifier.

The chromosome-scale*R. chingii* genome reported terpene-synthase activity among expanded gene functions but did not provide a dedicated sequence-resolved TPS catalogue (Wang et al., 2021). Shi et al. (2024) subsequently analysed 172 *R. chingii* UGTs, demonstrating the value of family-level evidence for flavonoid glycosylation. Yet pathway-level KEGG orthology labels, family-specific Pfam profiles and coordinate-level GFF3 models can disagree, weakening attempts to connect food-quality chemistry to species-specific biosynthetic potential. Because aroma terpenes and flavonoid glycosides are two chemically relevant fruit-quality axes, we used TPS and UGT as independent annotation-audit cases after the maturity screen. Public developmental expression and near-relative genomes supplied orthogonal context, but neither was matched to the commercial lots; no geographic regulation, individual enzyme product or enzyme activity was inferred.

We assembled volatile, flavonoid-feature, amino-acid and electronic-sensing profiles across nine commercial dried-fruit groups and asked whether any chemical candidate remained outside the independently observed developmental envelope. Developmental-envelope coverage, residual range excess, leave-one-location-out analysis and Monte Carlo scenarios defined assumption-aware validation tiers. We then used the*R. chingii* reference genome, public developmental RNA-seq and two near-relative genomes to audit UGT and TPS families linked to flavonoid glycosides and aroma terpenes and to evaluate abnormal gene-model boundaries. The study therefore delivers a two-gate prioritisation framework while keeping observed lot differences, candidate marker status and validated provenance inference as separate evidence levels.

## 2 Materials and methods

### 2.1 Samples and reagents

Nine*R. chingii* groups comprised seven sampling groups across six Zhejiang county-level areas plus two Anhui counties, including cultivated and wild material from Pan'an (Table 1). The chemical data analysed here were not generated de novo for the present study; they were compiled as location-level summaries from previously produced vendor and laboratory reports (one summary column per group for the volatile and LC-MS/MS tables, one submitted profile per group for the amino-acid reports, and a technical-repeat export for the electronic-nose record). Historical sampling records referred to three biological replicates per group, but because replicate-level observations were not preserved and their aggregation cannot be reconstructed, plant-level sampling, pooling across farms or plants, harvest date, genotype, maturity criteria, drying and storage history cannot be resolved. Samples were identified as dried *R. chingii* fruits by Hangzhou TCM Hospital. These metadata limits define the scope of the location-level interpretation.

**Table 1:** Sample information and microenvironmental factors of*Rubus chingii.*

Sample No.	Producing Area (Province–City/County)	Type	Altitude (m)	Soil pH	Annual Sunshine Hours (h)
F1	Anhui–Bozhou	Cultivated	42	6.8	1750
F2	Zhejiang–Taizhou (Yuanqiao)	Cultivated	85	6.2	1820
F3	Zhejiang–Lishui (Jinyun)	Cultivated	320	5.4	1780
F4	Zhejiang–Shaoxing (Shengxian)	Cultivated	98	6.0	1850
F5	Zhejiang–Taizhou (Sanmen)	Cultivated	55	6.3	1790
F6	Anhui–Xuancheng (Guangde)	Cultivated	110	6.5	1760
F7	Zhejiang–Wenzhou (Yongjia)	Cultivated	480	5.2	1880
F8	Zhejiang–Jinhua (Pan'an)	Cultivated	650	5.6	1920
F9	Zhejiang–Jinhua (Pan'an)	Wild	720	5.3	1950

Reagents included 2-octanol internal standard, 18 amino-acid standards, rutin and quercetin standards, and chromatographic-grade solvents. The available records identify an Agilent 8890-5977B GC–MS for the volatile profile, a Shimadzu LC-2030PLUS HPLC system for the compiled amino-acid reports and a Waters e2695 HPLC-UV system; the sensing and LC-MS/MS platforms are specified with their respective analyses below, and exact configurations not present in the available records are not inferred.

GIS environmental factors were summarised as location-level averages for altitude, soil pH and annual sunshine hours over the preceding five years. The compiled dataset does not preserve the source raster, spatial resolution, extraction coordinates or whether each value represents an individual site or a production-area mean. Rainfall, temperature, soil nutrients and agricultural practices were not available.

### 2.2 Experimental methods

#### 2.2.1 Volatilomics analysis (HS-SPME-GC–MS)

One gram of ground sample was placed in a 20-mL headspace vial with saturated NaCl and 2-octanol internal standard; volatile profiling was performed by Jiaxing Metware Biotechnology Co., Ltd. using HS-SPME (DVB/CAR/PDMS fibre, 60 °C extraction) with GC–MS on a DB-5MS column in EI mode (*m*/*z* 50–500), with full instrument parameters in Supplementary Methods S1. No project-specific optimisation experiments are documented; the protocol follows the vendor's standardised commercial service procedure, and no optimisation history beyond this standard protocol was available. The dataset reports NIST 17 library matching with retention-index support and internal-standard quantification. Retention-index values, reference-standard chromatograms, raw spectra and a complete match ledger were unavailable. All 124 volatile rows are therefore treated as library/RI-supported putative annotations; no volatile is designated standard-confirmed.

#### 2.2.2 Electronic-nose analysis

Measurements used a SuperNose electronic nose (Shanghai Ruifen Intelligent Technology Co., Ltd.) with an array of 14 metal-oxide-semiconductor sensors. Twenty grams of sample were sealed in the sample vial and allowed to equilibrate for at least 30 min at controlled ambient temperature and humidity, and 10 mL of headspace gas was injected per measurement. Clean air served as carrier gas at 1 L/min, with a 2 L/min cleaning flow applied for at least 30 s until baseline stabilisation, a 25 s wait time and a detection temperature of 25 °C. Four parallel measurements per sample were recorded for each of the nine groups; the report states that outliers had been removed before analysis. These measurements were used to describe sensor repeatability and to generate a technical-repeat PCA, not as independent biological or geographic observations. The 14-sensor array covers manufacturer-handbook target classes spanning amines, sulfides, organic solvents, ketones/alcohols/aldehydes and flammable gases (instrument-handbook description as published in Wang et al., 2023). Short-term drift control followed the standard operating cycle of clean-air flush and automated baseline reset between injections (Rock et al., 2008; Rudnitskaya, 2018).

#### 2.2.3 Electronic-tongue analysis

Measurements used a SmarTongue electronic tongue (Shanghai Ruifen Intelligent Technology Co., Ltd.) with six inert-metal sensors (platinum, gold, palladium, titanium, tungsten and silver) operated by voltammetric pulse excitation. Equal sample volumes were measured after sensor preheating and cleaning. Four parallel measurements per sample were recorded for each of the nine groups; the report states that outliers had been removed before analysis. The available report provided ANOVA, pairwise-comparison (Tukey), rendered PCA/LDA and Euclidean-clustering outputs for the nine groups, but no exportable sensor-response matrix, and the aqueous sample-preparation conditions before measurement were not specified in the report. These report-derived outputs are shown as descriptive instrument summaries and are not reanalysed as a sample-level geographic test.

#### 2.2.4 Amino-acid analysis

Homogenised material was hydrolysed under nitrogen with 10 mL of 1:1 hydrochloric acid containing 1% phenol at 110 °C for 24 h. A 5-mL aliquot of the cooled, filtered hydrolysate was evaporated, reconstituted in 1 mL of 0.1 mol/L hydrochloric acid and filtered through a 0.22-μm membrane before pre-column derivatisation with o-phthalaldehyde/3-mercaptopropionic acid (reagent A) and FMOC (reagent B). Separation used a Shimadzu LC-2030PLUS with a Diamonsil-plus C18-A column (4.6 × 250 mm, 5 μm) with dual-wavelength detection (338/262 nm); gradient and mobile-phase compositions are given in Supplementary Methods S1. Analyses were performed by the Plant Laboratory of Beijing ZKGX Research Institute of Chemical Technology (report IDs ZX210624–240101 to ZX210624–240109, dated 14 July 2021). Nine laboratory reports, one per location group, provided 18 amino-acid results in mg/kg submitted dried material. Tryptophan was reported as not detected in all nine reports and was excluded from nutritional interpretation because acid hydrolysis does not provide a valid estimate without a separate validated procedure. Reported glutamate and aspartate are interpreted as combined Glu + Gln and Asp+Asn pools because acid hydrolysis converts glutamine and asparagine. Moisture correction was not documented, so values are not relabelled as mg/kg dry weight.

#### 2.2.5 Flavonoid and LC-MS/MS feature analyses

For the targeted HPLC-UV method, 0.5 g of sample was extracted with 20 mL of 70% methanol under ultrasonication at 40 °C for 30 min (200 W), followed by centrifugation and 0.22-μm filtration. Separation used a C18 column (4.6 × 250 mm, 5 μm) with a methanol/0.1% formic acid gradient (programme in Supplementary Methods S1) at 1.0 mL/min and 30 °C. The method included 12 characteristic peaks at 360 nm and used rutin and quercetin standards. Separately, the LC-MS/MS dataset was acquired by Jiaxing Metware Biotechnology Co., Ltd. on the Nexera X2–4500 QTRAP platform in MRM mode (Agilent SB-C18 column, 1.8 μm, 2.1 × 100 mm; water and acetonitrile each with 0.1% formic acid; Analyst 1.6.3 and MultiQuant processing against the MWDB database); freeze-dried powder (100 mg) was extracted with 1.2 mL of 70% methanol, vortexed intermittently, kept at 4 °C overnight, centrifuged and 0.22-μm filtered. The LC-MS/MS table contained 318 location-level feature rows with precursor/product ions, retention-time/database matching levels, nine group columns and three pooled-QC columns. Level 1 and Level 2 labels are reported as putative evidence tiers because raw spectra and fragment-match scores were not available. Only results with explicit authentic-standard support are described as standard-confirmed.

### 2.3 Data structure, preprocessing, evidence tiers and statistical analysis

Each analytical layer was processed separately. Volatile and LC-MS/MS matrices were summarised by location group, amino-acid reports were treated as one submitted profile per location, electronic-nose measurements were treated as technical repeats and electronic-tongue outputs were treated as report-derived summaries. Both chromatographic tables included three pooled-QC columns, and the amino-acid reports covered 17 quantified analytes with tryptophan reported as not detected. These columns represent location-level summaries rather than a reconstructable sample-level matrix. No replicate variance, sample-level ANOVA, VIP ranking or geographic classifier was re-estimated from the location-summary matrices. Supplementary Tables S1-S5 provide the candidate inventory, future-model requirements, amino-acid source matrix, derived compositional outputs and independent-unit ledger in numerical order. Public developmental data and reference genomes were analysed as independent context layers, not as matched geographic validation.

Accordingly, chemical rankings and cross-platform patterns were treated as descriptive observations. Technical repeats were used only to assess measurement repeatability. Inferential location contrasts, supervised origin classification and causal environmental attribution were not estimated from the summary matrices.

Candidate-feature selection and classifier estimation were not repeated because the independent sample-level matrix and fold-specific preprocessing objects were unavailable. The 18-entity candidate list is therefore used only for assay prioritisation.

No geographic classifier was fitted to the location-summary matrices. Future-model matrix requirements are specified in Supplementary Table S2.

Environment-metabolite associations were evaluated with the nine locations as independent units. Three prespecified comparisons were tested using Spearman correlation, exact enumeration of all 9! = 362,880 label permutations, 10,000 bootstrap resamples and Benjamini-Hochberg correction. Province and altitude were treated as structurally confounded because both Anhui locations were low altitude. ANOVA, OPLS-DA, LDA and classification were not included because their independent-unit matrices and resampling objects were unavailable. All reported association*P* values are permutation-based and corrected across the three tests.

### 2.4 Public developmental metabolite and full RNA-seq evidence integration

The public NCBI project PRJNA671545 was used as an independent developmental-context resource. The reconciled design included 12 paired-end fruit RNA-seq libraries, three biological replicates at each of four publication-defined stages (big green, green to yellow, yellow to orange and red). NCBI BioSample labels reversed YO and Re for six libraries; publication definitions and sample-name prefixes were used for analysis, while the discrepancy was retained in the design table. The publication-associated developmental metabolite table contained 24 samples (six per stage) and 21 features. It was reanalysed separately using log2-transformed values, Kruskal-Wallis statistics and Benjamini-Hochberg correction. The fresh-fruit public values were not pooled with the commercial dried-fruit location dataset.

For each of the 11 name- or formula-level overlaps, the nine-location log2 range (Rloc) and four-stage developmental log2 range (Rdev) were compared. Developmental-envelope coverage was*C* = min(Rdev/Rloc, 1), and residual range excess was *E* = max(Rloc - Rdev, 0). Positive E values identify candidates whose location range exceeds the observed developmental envelope; C is a bounded range comparison, not a causal attribution fraction. Robustness was assessed by deleting each location once and recalculating E. Scenario sensitivity was evaluated with 30,000 Monte Carlo runs per setting. Each run generated nine synthetic lots from Dirichlet stage mixtures (α = 0.5, 1 or 2), bootstrapped the six public observations per stage and applied an assumed lot-level drying factor on the log2 scale (SD = 0, 0.25 or 0.5). Tier 1 required positive full-data E, retention in at least 8/9 leave-one-location-out analyses and an observed range above the simulated q95 in all three settings at drying SD = 0.25. Tier 2 required positive full-data E and retention in at least 8/9 analyses but did not meet the Tier 1 scenario criterion. Because stage mixtures and drying SDs were not measured, these outputs are scenario analyses rather than parameter estimates.

Stage-level expression-metabolite coupling was audited across 247 unique candidate genes and 21 metabolites. Genes with a maximum stage mean of at least 0.1 TPM and a non-constant four-stage trajectory were tested. Spearman correlations used log2(TPM + 0.1) and log2 metabolite stage means. Two-sided*P* values enumerated all 24 stage permutations and were adjusted across all tested gene-metabolite pairs by the Benjamini-Hochberg method. The RNA-seq and metabolite samples were not matched, and *n* = 4 stage means gives a minimum attainable exact two-sided P of 0.0833; this analysis can nominate trajectories but cannot establish regulation. Supplementary Tables S6-S8 contain the range-score, coupling-audit and public-gene outputs in numerical order.

All FASTQ files were required to match the ENA-reported byte counts before processing. Read quality and adapter contamination were assessed for every library with FastQC and trimmed with Trimmomatic (parameters in Supplementary Methods S1) before alignment. Reads were then aligned to the *Rubus chingii* Hu v1.0 reference with Subjunc 2.0.2 in paired-end splice-aware mode; annotated transcript exons were quantified with featureCounts 2.0.3 in paired fragment-counting mode (orphan and chimeric pairs excluded). Raw counts entered DESeq2 with stage contrasts adjusted over the full quantified transcriptome, and exon-length-normalised TPM was generated for visualisation. Candidate-level Kruskal-Wallis tests were treated as secondary prioritisation statistics. Exact annotated-junction support was independently summarised for LG03.2235 and LG02.1599 across all 12 libraries, with the longest model intron reported separately from overall junction recovery. The Subjunc/Subread and featureCounts procedures follow Liao, Smyth and Shi, 2013, Liao, Smyth and Shi, 2014, and transcriptome-wide differential analysis follows Love et al. (2014).

### 2.5 Reference-genome resources, functional annotation and sequence anchoring

The public*Rubus chingii* Hu v1.0 reference-genome resources were obtained from the Genome Database for Rosaceae; the assembly and annotation version follow the chromosome-scale reference genome paper (Wang et al., 2021), and the corresponding accessions are recorded in the Data availability section. The analysis included 33,130 predicted protein-coding sequences, 6880 KEGG orthology records representing 6861 genes and 22,248 pathway records representing 8463 genes. Eight KEGG pathways were prespecified: phenylpropanoid biosynthesis, flavonoid biosynthesis, terpenoid backbone biosynthesis, monoterpenoid biosynthesis, alanine/aspartate/glutamate metabolism, valine/leucine/isoleucine biosynthesis, valine/leucine/isoleucine degradation and nitrogen metabolism. The two branched-chain-amino-acid pathways were merged only for visualisation, yielding seven groups.

Pathway genes were counted after deduplication by reference-gene identifier. Candidate biosynthetic genes were defined using KEGG orthology terms for flavonol synthase, dihydroflavonol 4-reductase, flavonol glycosyltransferases, anthocyanidin glycosyltransferases, glutamate dehydrogenase, and aspartate aminotransferase. Linkage-group distributions were calculated across LG01–LG07. Hypergeometric tests with Benjamini–Hochberg correction were used to screen for linkage-group enrichment. This analysis identifies genomic pathway capacity and candidate loci, not expression differences in the current locations.

Complete public coding sequences for*RcFLS1*, *RcFLS2*, *RcDFR*, *RcUGT75L20*, *RcUGT75T4*, and *RcUGT85A57* were translated and mapped without restricting the search to prespecified KEGG orthology classes. The six public coding sequences (*RcFLS1*, *RcFLS2*, *RcDFR*, *RcUGT75L20*, *RcUGT75T4* and *RcUGT85A57*) used as mapping queries are listed in Supplementary Table S8. For each query, shared 7-mers were used to screen all 33,130 *Rubus chingii* Hu v1.0 predicted proteins, the 25 highest-recall candidates were globally aligned, and hits were ranked by amino-acid identity multiplied by query coverage. This two-stage design uses the shared-7-mer seed screen as a fast, sensitive pre-filter over the 33,130-protein proteome and restricts global alignment to the 25 highest-recall candidates to keep the procedure tractable while retaining near-optimal homologues; the identity and coverage thresholds follow standard practice for recognising orthologous homologues. Mappings with at least 90% identity and 95% query coverage were designated high confidence; mappings with at least 80% identity and 75% query coverage were considered provisional. Reference/query length asymmetry was recorded to flag possible truncated or fused gene models. *RcDFR* and *RcUGT75L20* were classified as provisional when coverage was below 95%, even if identity was high. All high-confidence and provisional mappings were independently confirmed by BLASTP top-hit search (Camacho et al., 2009) against the same *Rubus chingii* Hu v1.0 proteome, and only mappings whose BLASTP top hit was concordant with the seed-based hit were retained. This sequence analysis assigns public genes to reference loci but does not quantify expression in the nine locations.

### 2.6 Family-level annotation audit of UGT and TPS candidates

For the UGT audit, all 33,130 *Rubus chingii* Hu v1.0 predicted proteins were scanned with the PF00201 UDP-glycosyltransferase profile using PyHMMER 0.12.1, with sequence- and domain-level *E*-value thresholds of 1 × 10^−5^. All significant PF00201 hits were included as the operational family catalogue; unless stated otherwise, all UGT model counts in this manuscript refer to this PyHMMER 0.12.1 baseline. A separate C-terminal profile-compatibility flag recorded hits ending within 60 amino acids of the protein C terminus; this flag was not used to exclude family members. KEGG descriptions containing glucosyltransferase, glucuronosyltransferase, rhamnosyltransferase, xylosyltransferase or phlorizin synthase were labelled UGT-related, while other KO labels and missing labels were included as discordant or absent annotations. Shi et al. (2024) built the 172-member RchUGT catalogue from this same genome using a family-profile (HMM) approach; because our scan applied the same family-profile logic, recovering all of those loci is an expected consistency check rather than an independent finding. The operational catalogue was therefore compared locus by locus with that published catalogue; overlap and additional profile-defined candidates were reported without assigning substrate specificity. Profile searches and family definitions followed PyHMMER/HMMER and Pfam conventions (Eddy, 2011; Larralde & Zeller, 2023; Mistry et al., 2021).

#### 2.6.1 Species-matched positive-control benchmark and detection-route comparison

*RcUGT75L20*, *RcUGT75T4* and *RcUGT85A57*, three publicly deposited and experimentally cloned *R. chingii* UGT coding sequences (Huang et al., 2024), were used as a small species-matched positive-control set. Their recovery was compared among the prespecified KO-only flavonol-glycosyltransferase screen, the whole-proteome PF00201 scan and the intersection of PF00201 hits with UGT-related KO labels. To place three-of-three PF00201 recovery against the proteome background, 10,000 sets of three proteins were sampled without replacement from the predicted proteome using random seed 20,260,723. The statistic was the number of PF00201 hits per set. An exact upper-tail hypergeometric probability was calculated as the primary benchmark; the plus-one empirical *P* value from 10,000 random sets was included as a secondary finite-simulation check. This benchmark evaluates recovery sensitivity for three known species-matched controls; it does not estimate functional specificity, substrate-level precision or the proportion of the 178 candidates that are active flavonoid UGTs.

Because family-profile, orthology and motif routes can disagree, we compared three executable routes on the same proteome. Route 1, whole-proteome PF00201 HMM scanning (PyHMMER 0.12.3, E ≤ 1 × 10^−5^), identified 189 models (179 at Pfam gathering cutoffs; 173C-terminally complete). Route 2, KEGG orthology, annotated 6861 genes; the prespecified flavonol-specific K22794 screen alone yields 6 genes, and 58 of the 189 PF00201 models carry UGT-related KO labels while 129 carry none. Route 3, a PSPG-motif regular-expression route (PROSITE PS00375 consensus), returned zero proteins in this proteome and was abandoned; dedicated PSPG-profile, InterPro and eggNOG comparisons are deposited as a turnkey pipeline for local completion. Relative to the original scan (178 models, PyHMMER 0.12.1), the small drift is attributable to Pfam/PyHMMER version updates and does not change the direction or significance of any downstream result.

#### 2.6.2 Structural prioritisation of UGT candidates

Beyond sequence-level family assignment, we asked whether candidate UGTs present binding pockets structurally compatible with the prioritised compounds of Table 2. The 173C-terminally complete PF00201 models were screened for template support against eight plant UGT crystal structures (phmmer; template accessions in Supplementary Methods S1); 124 candidates exceeded 35% sequence identity to a template. Thirty candidates meeting pre-registered criteria (template identity ≥ 35%, coverage ≥ 0.5, a cap of three per tandem cluster, and obligatory inclusion of the RcUGT85A57 locus LG03.1781) were modelled as their C-terminal catalytic domains (350 aa) with ESMFold; 29 of 30 models reached pLDDT ≥ 86 (one fragmentary model, LG03.3408, is flagged and excluded from ranking). Each model was docked with the four aglycone acceptors (kaempferol, quercetin, gallic acid and ellagic acid) using AutoDock Vina with the search grid centred on the PSPG-box tryptophan. Docking provides structural compatibility rankings, not evidence of catalytic activity or substrate specificity; experimental enzyme assays remain the definitive test. The phylogenetic placement and structural-compatibility ranking of the prioritised candidates are shown in Supplementary Figs. S1 and S2, and the full ranking with model quality metrics is deposited with the Supplementary Data and code archive. Conversely, the RcUGT75T4 locus (LG07.1014) fell below the pre-registered modelling thresholds (31.3% identity, 45% coverage) and was excluded; this negative result is reported rather than rescued post hoc.

**Table 2:** Developmental-envelope tiers linking commercial-fruit chemistry, robustness and genomic context. Tier 1 requires positive full-data range excess, retention in at least 8/9 leave-one-location-out analyses and exceedance of the scenario q95 in all three moderate drying settings; Tier 2 meets the first two criteria but not the third. Shared requirements are authentic-standard or method-appropriate confirmation and independent stage-controlled commercial lots. No row is an established geographic marker.

Candidate	Chemical evidence	Developmental evidence	Genomic context	Developmental-envelope tier and next test
Gallic acid	putative LC-MS/MS; local log2 range 3.40	BH*q* = 0.00522; developmental log2 range 3.00	hydrolysable-tannin pathway context (gallic acid and its derivatives; Wang et al., 2021)	Tier 1 (*E* = 0.41; LOO 8/9; moderate scenarios 3/3); standard confirmation and stage-controlled replication
Naringenin chalcone	putative LC-MS/MS; local log2 range 1.36	BH*q* = 0.00058; developmental log2 range 2.16	sequence-anchored FLS/DFR flavonol-pathway context	Development-sensitive descriptor; retain for stage-controlled quality profiling
Naringenin	putative LC-MS/MS; local log2 range 1.99	BH*q* = 0.00058; developmental log2 range 2.16	sequence-anchored FLS/DFR flavonol-pathway context	Development-sensitive descriptor; retain for stage-controlled quality profiling
Quercetin	putative LC-MS/MS; local log2 range 1.15	BH*q* = 0.000997; developmental log2 range 2.01	sequence-anchored FLS/DFR flavonol-pathway context	Development-sensitive descriptor; retain for stage-controlled quality profiling
Rutin	putative LC-MS/MS; local log2 range 0.95	BH*q* = 0.000997; developmental log2 range 1.54	sequence-anchored FLS/DFR context plus PF00201 UGT catalogue (candidate glycosylation locus)	Development-sensitive descriptor; retain for stage-controlled quality profiling
Gallotannin	putative LC-MS/MS; local log2 range 1.15	BH*q* = 0.0438; developmental log2 range 1.49	hydrolysable-tannin pathway context (gallotannin branch; Wang et al., 2021)	Development-sensitive descriptor; retain for stage-controlled quality profiling
Dihydroquercetin	putative LC-MS/MS; local log2 range 0.69	BH*q* = 0.000708; developmental log2 range 1.40	sequence-anchored FLS/DFR flavonol-pathway context	Development-sensitive descriptor; retain for stage-controlled quality profiling
Dihydrokaempferol	putative LC-MS/MS; local log2 range 1.01	BH*q* = 0.00129; developmental log2 range 1.29	sequence-anchored FLS/DFR flavonol-pathway context	Development-sensitive descriptor; retain for stage-controlled quality profiling
Kaempferol	putative LC-MS/MS; local log2 range 1.18	BH*q* = 0.000997; developmental log2 range 1.27	sequence-anchored FLS/DFR flavonol-pathway context	Development-sensitive descriptor; retain for stage-controlled quality profiling
Kaempferol 3-O-glucoside	putative LC-MS/MS; local log2 range 1.30	BH*q* = 0.00325; developmental log2 range 0.92	sequence-anchored FLS/DFR context plus PF00201 UGT catalogue (candidate glycosylation locus)	Tier 2 (*E* = 0.38; LOO 9/9; moderate scenarios 1/3); standard confirmation and stage-controlled replication
Ellagic acid	putative LC-MS/MS; local log2 range 1.49	BH*q* = 0.0143; developmental log2 range 0.57	hydrolysable-tannin pathway context (ellagitannin-derived; Wang et al., 2021)	Tier 1 (*E* = 0.92; LOO 9/9; moderate scenarios 3/3); standard confirmation and stage-controlled replication
Linalool	putative GC–MS/RI; highest YQ, lowest BZ	not present in the 21-metabolite public table	TPS-b/g branch context; LG03 TPS concentration	Aroma priority; standard/RI confirmation and stage-controlled replication
Benzaldehyde	putative GC–MS/RI; highest SX, lowest PA	not present in the 21-metabolite public table	explicitly excluded from TPS product linkage	Aroma priority; standard/RI confirmation and stage-controlled replication
Glutamate pool	acid-hydrolysis report; interpreted as Glu + Gln	no matched public developmental measurement	GDH/AAT pathway context only	Composition priority; free-amino-acid assay, moisture basis and stage-controlled replication
Aspartate pool	acid-hydrolysis report; interpreted as Asp+Asn	no matched public developmental measurement	GDH/AAT pathway context only	Composition priority; free-amino-acid assay, moisture basis and stage-controlled replication

For the TPS audit, the *Rubus chingii* Hu v1.0 proteins were screened with the Pfam TPS domains PF01397 and PF03936, followed by catalytic-motif review. The operational motif-supported set required a C-terminal TPS domain and a class-I or class-II catalytic signature. Candidate definitions and thresholds are provided in Supplementary Methods S1.

Candidate loci were cross-referenced with GDR KO records, and linkage-group concentration was tested against the full proteome using one-sided hypergeometric tests with Benjamini-Hochberg correction. Domain and motif evidence was used for family candidacy, not product assignment.

### 2.7 GFF3 coordinate audit and phylogeny-anchored TPS analysis

TPS candidates were mapped to*Rubus chingii* Hu v1.0 GFF3 gene, transcript, exon and CDS records. Coordinate overlap was treated as a model-boundary flag rather than evidence of tandem duplication. Detailed coordinate rules are provided in Supplementary Methods S1.

For phylogenetic context, 32 motif-supported candidates were analysed with 25 functionally characterised TPS references obtained from curated UniProt records (UniProt Consortium, 2023); the characterised reference set is summarised in Supplementary Table S8. Multiple-sequence alignment used MAFFT (L-INS-i mode; Katoh et al., 2019), alignment columns were trimmed with ClipKIT (default gap threshold; Steenwyk et al., 2020), the best-fit substitution model was selected with ModelFinder (Kalyaanamoorthy et al., 2017), and a maximum-likelihood tree was inferred with IQ-TREE using 1000 ultrafast bootstrap replicates (UFBoot2; Hoang et al., 2018; Minh et al., 2020), with rooting anchored on the functionally characterised reference clades. A high-resolution rendering with legible UFBoot2 branch-support values and a scale bar is provided as Supplementary Fig. S3. The tree provides branch-level context; the alignment and tree files are provided in the Supplementary Data, and the subfamily assignment of all 32 candidates is given in Supplementary Table S8. As a robustness check under current Pfam/PyHMMER versions, the motif-supported screen was repeated with identical rules, yielding 47 candidates with the same LG03 concentration, and a Rosaceae-expanded reference set of 69 proteins (39 *R. occidentalis* two-domain TPS, 5 characterised Rosaceae TPS and 25 characterised references) was aligned with MAFFT and analysed with IQ-TREE under LG + R4; the alignment, tree file and per-candidate branch placement are deposited with the Supplementary Data.

For comparative boundary review, LG03.2235 and LG02.1599 proteins were divided at the N-terminal/TPS transition and searched with NCBI BLAST+ 2.17.0 (Camacho et al., 2009). TBLASTN used the chromosome-scale *Rubus idaeus* (red raspberry, cultivar ‘Varnes’) assembly NIAB_RidaVarnes_1.0 (GenBank GCA_047496405.1), with flanking *R. chingii* proteins used to define the local homologous interval. BLASTP used the *Rubus occidentalis* (black raspberry) v3.0 protein catalogue and GFF3 coordinates (VanBuren et al., 2018). Segment placement was interpreted as split-model support only when the N and C segments occupied non-overlapping homologous blocks or separate annotated proteins. Public long-read searches distinguished genomic WGS from transcript long-read evidence.

## 3 Results

### 3.1 Multi-platform chemical profiles across nine producing locations

#### 3.1.1 Volatile profiles

The compiled volatile table contained 124 feature rows across nine location groups, including 107 named volatile entries represented in Fig. 1. Benzaldehyde was highest in SX (F4) and lowest in PA (F8), linalool was highest in YQ (F2) and lowest in BZ (F1), and the reported (Z)-2-heptenal feature was highest in SX (F4) and lowest in SM (F5) (Table 3). The E/Z assignment was not considered definitive because retention-index values, raw spectra and a complete match ledger were unavailable. These profiles describe location-level chemical variation; no SD, ANOVA *P* value or *q* value was inferred from the summary columns.

**Fig. 1:**
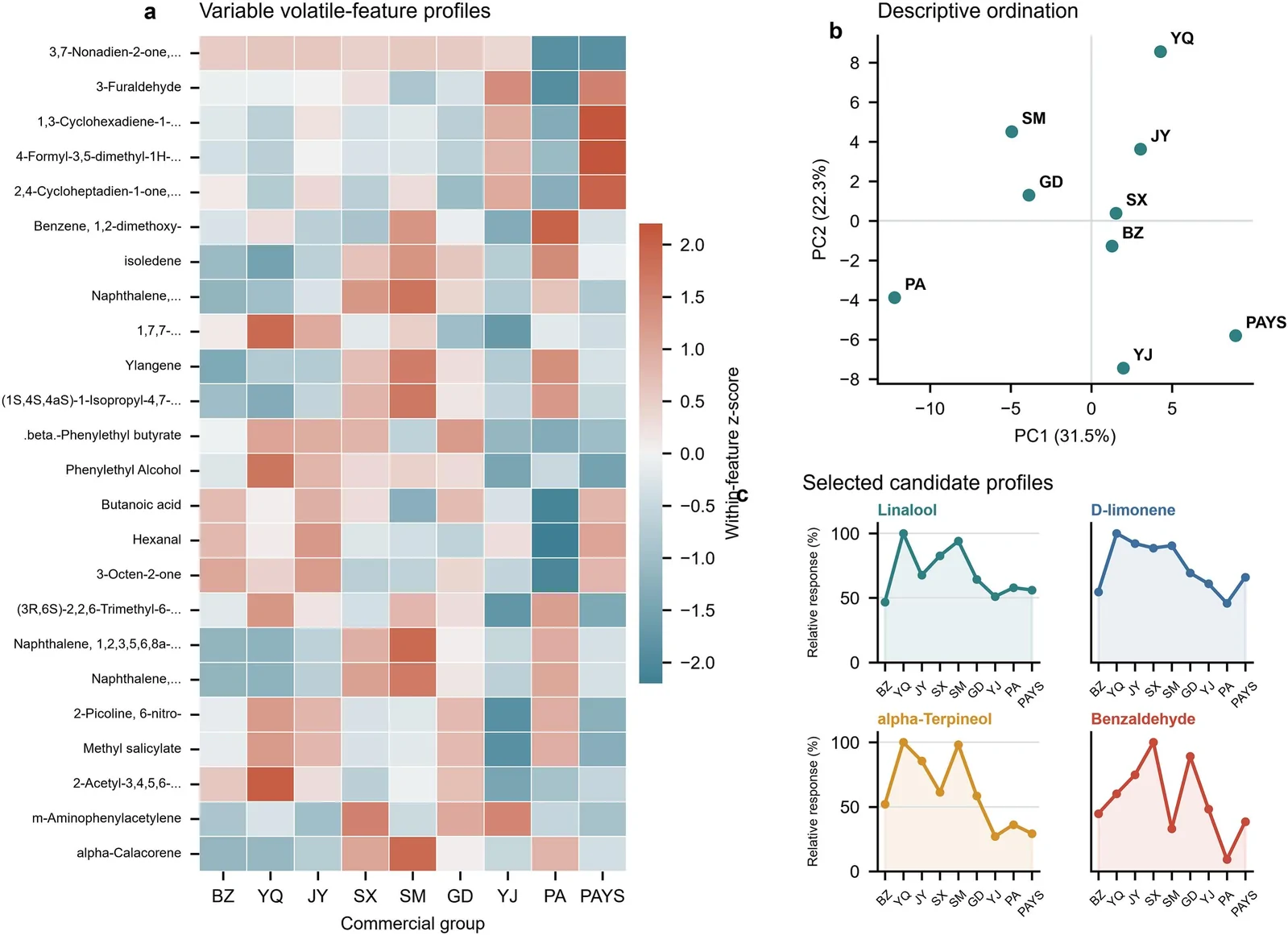
Volatile profiles of*R. chingii* fruit across nine producing-location groups. The panels show a hierarchical heatmap, a two-dimensional PCA score plot and representative volatile subclasses. The figure is descriptive: the underlying location columns do not provide biological-replicate variance or independent origin validation.

**Table 3:** Location-level extrema for selected volatile features (arbitrary reported intensity units; no replicate inference).

Reported feature	Annotation basis	Maximum location (F code)	Minimum location (F code)	Inference
Benzaldehyde	GC–MS library/RI annotation	SX (F4): 1,592,594	PA (F8): 145,177	Descriptive only;*n* = 9 location summaries
Linalool	GC–MS library/RI annotation	YQ (F2): 843,660	BZ (F1): 395,012	Descriptive only;*n* = 9 location summaries
2-Heptenal isomer (stereoisomer unresolved)	GC–MS library/RI annotation; stereochemistry not confirmed	SX (F4): 371,780	SM (F5): 37,429	Descriptive only;*n* = 9 location summaries

#### 3.1.2 Amino-acid composition

Nine laboratory reports provided one submitted dried-material profile for each location group (Fig. 2; Supplementary Tables S3-S4). Seventeen amino acids were quantified, whereas tryptophan was reported as not detected. The sum of the 17 quantified amino acids was highest in Yuanqiao F2 (83,537.9 mg/kg), followed by Pan'an cultivated F8 (80,973.1 mg/kg), and lowest in Pan'an wild F9 (63,751.2 mg/kg). Glutamic acid and aspartic acid were the dominant reported components, averaging 10,337.2 and 9765.5 mg/kg, respectively. F2 had the highest reported concentration for 10 of 17 analytes. These values describe submitted profiles rather than biological-replicate means. Glu + Asp, five sweet-associated amino acids and seven bitter-associated amino acids represented 24.8–30.0%, 23.5–25.8% and 35.5–39.0% of the quantified total, respectively. These are compositional groupings, not sensory intensities.

**Fig. 2:**
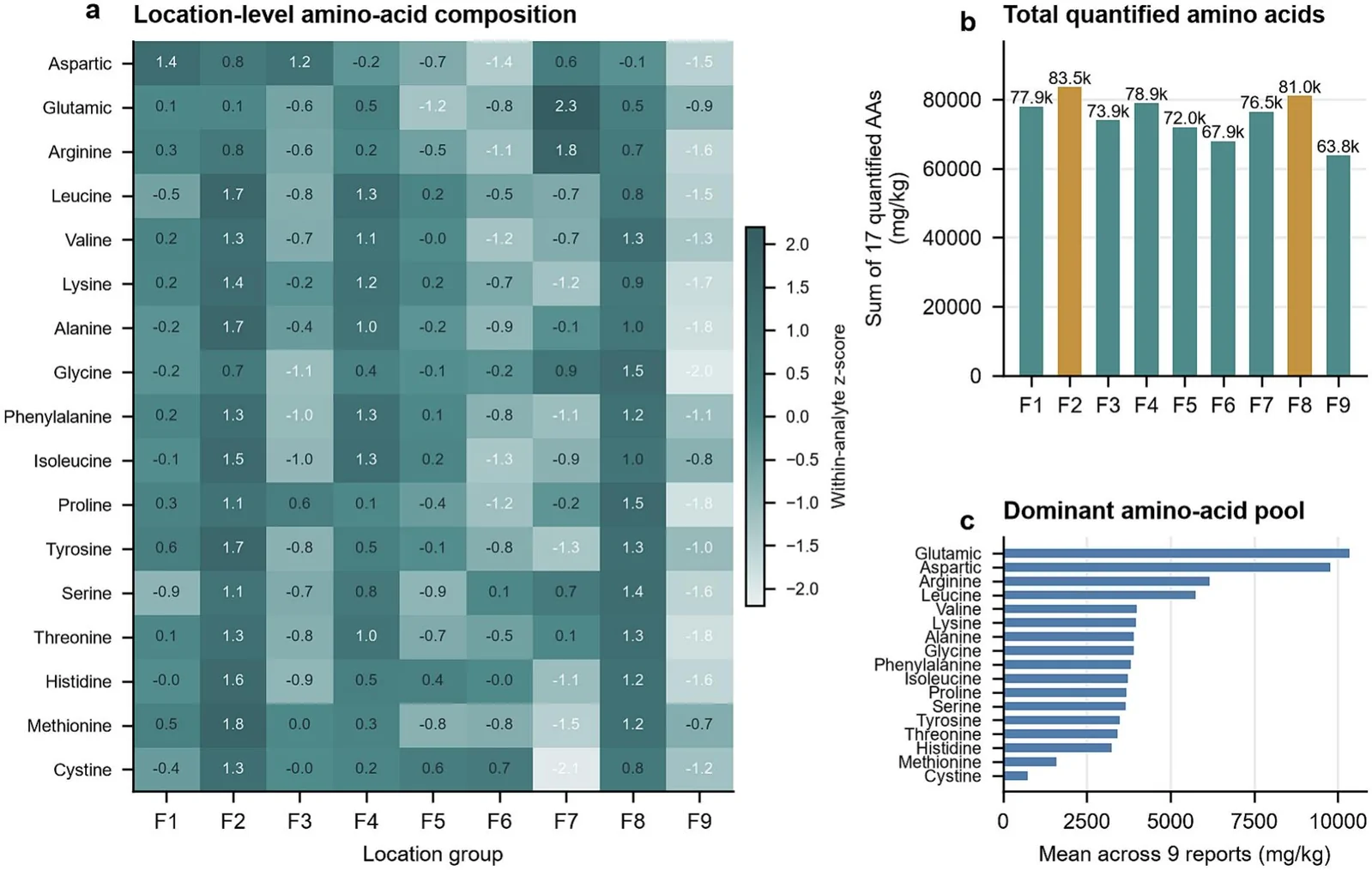
Location-level amino-acid composition of*R. chingii* fruit. The panels show within-analyte standardised profiles, summed quantified amino acids and dominant amino-acid pools across F1-F9. Each location is represented by one submitted dried-material report; no biological-replicate error bars or significance tests are shown.

#### 3.1.3 Flavonoid and LC-MS/MS feature profiles

The LC-MS/MS table contained 318 feature rows, distinct from the 12-peak HPLC-UV fingerprint (Fig. 3). Putatively annotated quercetin-3-O-rutinoside (rutin) was highest in PA (F8) and lowest in PA wild (F9), whereas the putative kaempferol aglycone feature was highest in YJ (F7) and lowest in SX (F4). These rankings are descriptive and are used to prioritize authentic-standard and targeted-MS confirmation. They are not presented as definitive compound identities unless standard support is explicit.

**Fig. 3:**
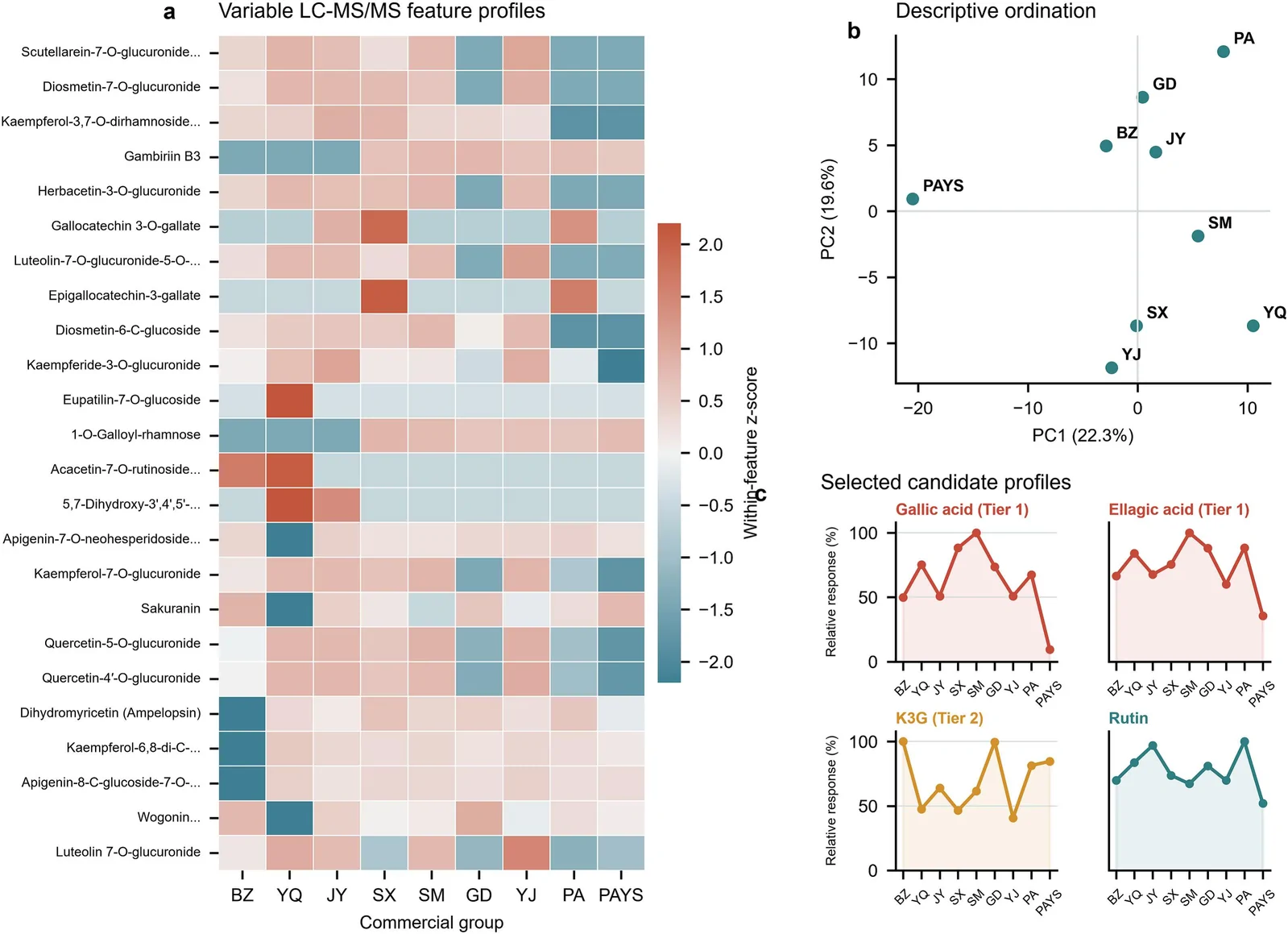
Flavonoid and LC-MS/MS feature profiles across producing-location groups. The panels show flavonol-feature contrasts, sample ordination, pathway-level context and subclass profiles. LC-MS/MS features are putatively annotated unless authentic-standard evidence is explicit; the figure does not establish definitive compound identity or geographic specificity.

#### 3.1.4 Electronic-nose and electronic-tongue profiles

The electronic-nose export contained four technical measurements per commercial group across 14 sensors. Sensor-level coefficients of variation within groups ranged from 0.10% to 9.28% (median 1.54%), quantifying instrumental repeatability, and group-mean sensor z-scores described the fingerprint structure (Fig. 4a–c). A PCA of the 36 technical measurements (PC1 50.2%, PC2 26.1%) reproduced the vendor report ordination exactly and placed the F9 centroid farthest from all other groups; the vendor report Tukey comparisons, retained as a report-level record, found no significant odour difference for five of 36 group pairs (F1-F4, F2-F5, F3-F6, F3-F8, F6-F7). These measurements are technical repeats and do not constitute biological replication. The vendor report outputs for the electronic tongue, retained as a report-level record, separated all nine samples: every one of the 36 Tukey pairs was reported as significant (least significant pair, samples 3–8, *p* = 0.018), the report PCA assigned 64.9% and 16.6% of variance to PC1 and PC2 with samples 3 and 5 closest, the report LDA assigned 54.7% and 33.2% with samples 2, 3 and 5 closest, and Euclidean clustering at the 0.6 scale grouped samples 8, 3 and 5, samples 1 and 4, and samples 9 and 7. The electronic-tongue response matrix itself was unavailable; therefore, the main figure reports the recoverable data-to-inference boundary rather than presenting report-derived *p* values as newly analysed evidence (Fig. 4d). The original lower-triangle significance categories are retained only as a transparent report-level record. Neither platform establishes a transferable origin classifier.

**Fig. 4:**
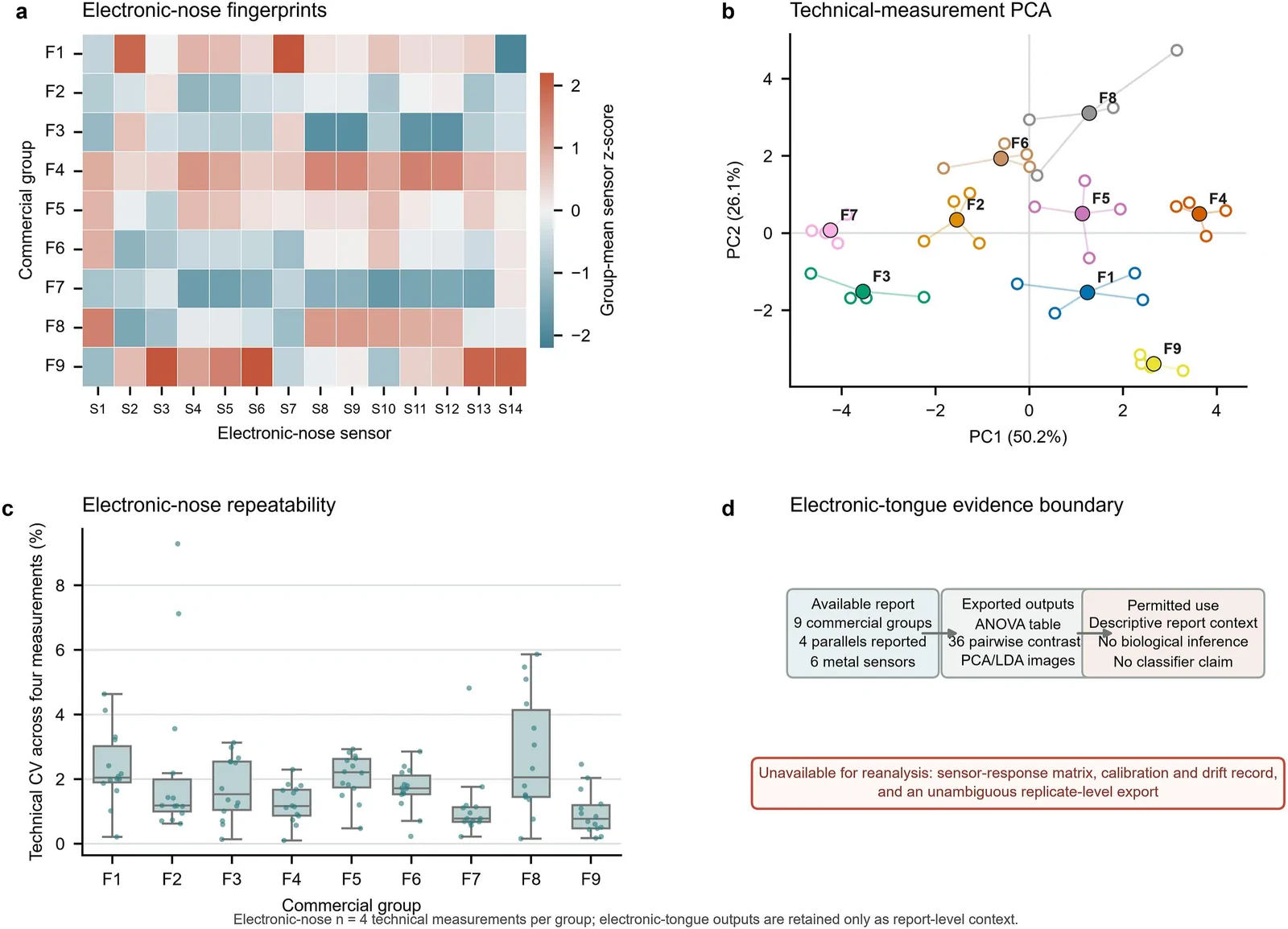
Electronic-sensing evidence and its inferential boundary across nine commercial groups. (a) Group-mean z-scores for 14 electronic-nose sensors. (b) Descriptive PCA of 36 electronic-nose technical measurements; points and group centroids expose within-group scatter. (c) Sensor-level coefficients of variation calculated from four technical measurements per group. These repeats are technical rather than biological. (d) Evidence flow for the electronic-tongue archive: the response matrix and unambiguous replicate definition were unavailable, so report-derived significance categories are retained only as a report-level record and are not used as independent geographic evidence or classifier validation.

### 3.2 Volatile and flavonoid features define a descriptive chemical landscape

The source feature list contained 20 labels, including the synonym pair quercetin-3-O-rutinoside/rutin. After synonym resolution, 18 non-redundant chemical entities remained (Supplementary Table S1). Because the sample-level matrix and fold-specific feature-selection objects were not available, the list is presented as a candidate inventory rather than a validated marker panel. The location maxima in the present feature tables are therefore used to guide targeted confirmation, not to claim marker specificity.

### 3.3 The available data support profiles, not inferential origin classification

No origin-classification performance is reported because the location-summary matrices do not contain independent sample-level observations. Supplementary Table S2 lists the sample matrix, grouped resampling, held-out predictions and calibration outputs required for a reproducible future evaluation.

### 3.4 Recorded environmental descriptors show no reproducible association at*n* = 9

Three prespecified environment-metabolite associations were evaluated at the location level (*n* = 9). These comparisons were prespecified against prior reports that altitude, temperature and light exposure can shift fruit phenolic and terpenoid profiles (Albert et al., 2009; Spitaler et al., 2006; Zoratti et al., 2014). Altitude was not associated with linalool (ρ = −0.317, bootstrap 95% CI −0.982 to 0.500, permutation *P* = 0.409) or rutin (ρ = 0.067, 95% CI −0.727 to 0.947, *P* = 0.882), and sunshine hours were not associated with linalool (ρ = −0.067, 95% CI −0.730 to 0.737, *P* = 0.880); all BH-adjusted *q* values were 0.882. With nine independent locations, approximate 80% power at a two-sided α of 0.05 is limited to correlations near |ρ| = 0.84, so moderate associations remain unresolved. Province and altitude are structurally entangled because both Anhui locations are low altitude whereas Zhejiang spans 55–720 m.

The available vectors did not support re-testing the soil-pH/glutamate or sunshine/total-terpenoid associations. These associations are not treated as verified results. The observed environmental descriptors are included as sampling metadata that can inform a crossed province-by-altitude design in future work.

### 3.5 Quality dimensions are reported as candidate priorities, not validated grades

The available integrated matrix did not permit reproducible entropy-weighted quality scoring or uncertainty analysis. We therefore do not assign location groups to validated quality grades. Instead, the chemistry, maturity and genome evidence are summarised as candidate priorities in Table 2, where each candidate is paired with the next measurement required for confirmation.

### 3.6 Public developmental transcriptomics provides expression context but no resolved metabolite coupling

The public RNA-seq alignment quantified 31,994 annotated models. Mapping was 96.2–97.0% per library, with 69.5–78.3% of input fragments assigned to annotated transcript models. The catalogue contained 247 unique TPS, UGT and sequence-anchored biosynthetic candidates. DESeq2 produced estimable stage contrasts for 152 expressed candidates; 104 passed full-transcriptome BH correction in at least one contrast, including 79/110 estimable UGT and 23/40 estimable TPS candidates. Stage-level coupling tested the 147 candidates with non-constant four-stage trajectories against 21 metabolites (3087 pairs). The exact two-sided *P* value could not be smaller than 0.0833, no pair passed BH correction and the minimum q was 0.411. Consequently, expression and metabolite trajectories remain parallel developmental priorities rather than evidence of gene-metabolite regulation.

The two long candidate models were transcribed in the public fruit libraries. LG02.1599 had 13 of 16 annotated junctions supported by 1114 exact cross-exon reads, while LG03.2235 had 21 of 26 supported by 1382 reads. Both models showed ordinary junction support in all 12 libraries. The longest annotated introns were 871 bp for LG02.1599 and 22,832 bp for LG03.2235, but the exact boundary-spanning junction for each received zero reads in every library. The evidence therefore supports partial transcript context with an unsupported critical model boundary, not a confirmed fusion or confirmed correction.

### 3.7 Developmental-envelope screening retains two tier 1 and one tier 2 candidate

The public developmental table contained 24 samples across four fruit stages, six per stage. Twenty of 21 metabolites showed a stage effect after Benjamini-Hochberg correction (q ≤ 6.2 × 10^−4^). All 11 name- or formula-level overlaps with the location dataset were stage-sensitive. Developmental ranges equalled or exceeded location ranges for eight overlaps, giving *C* = 1 and no residual range excess. Three candidates retained E > 0: gallic acid (Rloc 3.40, Rdev 3.00, E 0.41 log2 units), kaempferol 3-O-glucoside (1.30, 0.92, E 0.38) and ellagic acid (1.49, 0.57, E 0.92) (Fig. 5; Supplementary Table S6). Gallic acid retained positive E in 8/9 leave-one-location-out analyses; ellagic acid and kaempferol 3-O-glucoside did so in 9/9. Gallic acid and ellagic acid exceeded the scenario q95 in all three moderate drying settings and were assigned Tier 1, whereas kaempferol 3-O-glucoside exceeded q95 in 1/3 settings and was assigned Tier 2. The other eight overlaps were retained as development-sensitive descriptors rather than origin-marker candidates.

**Fig. 5:**
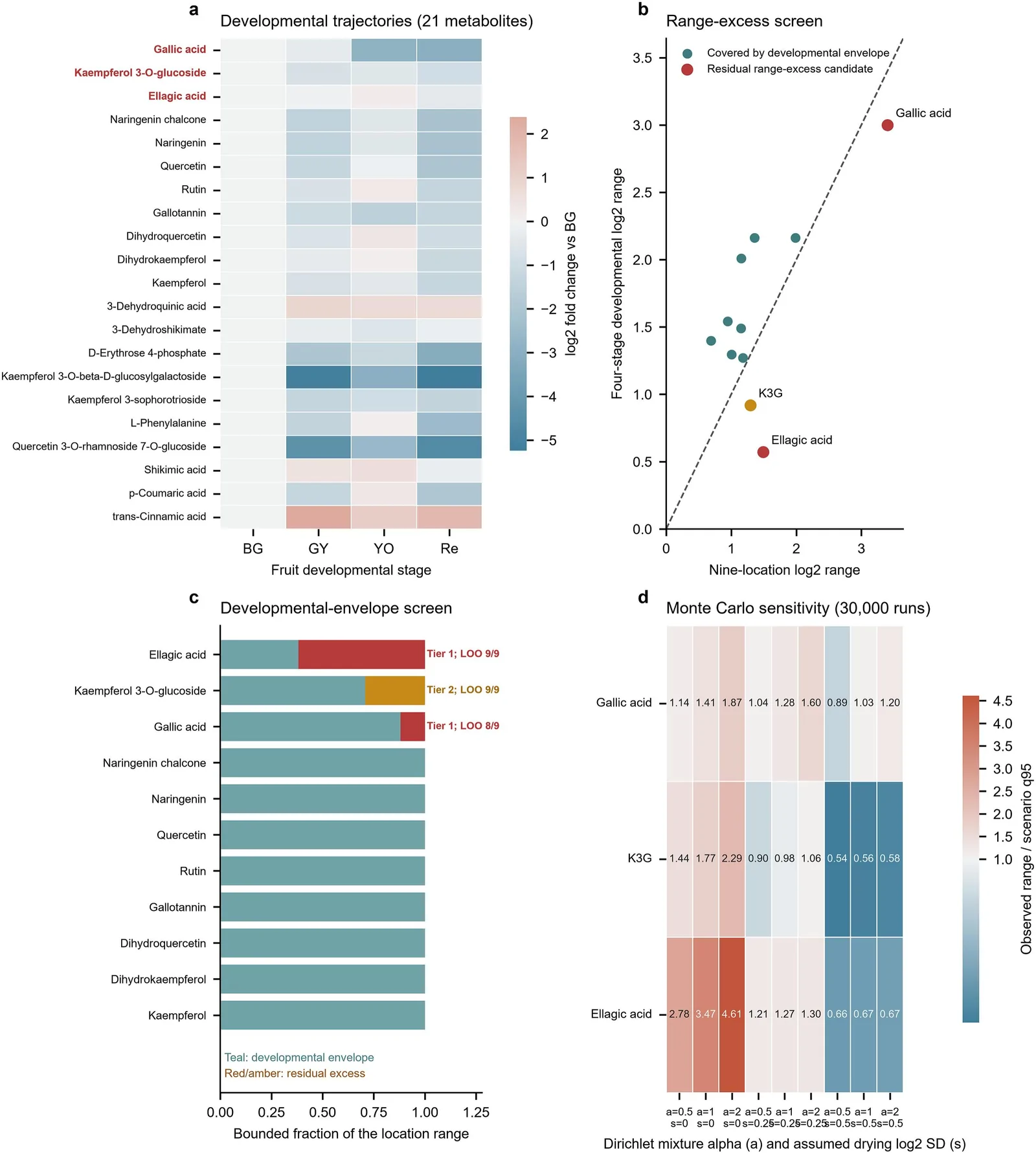
Developmental-envelope screening of commercial-fruit chemical candidates. (a) Four-stage trajectories for 21 public metabolites across 24 fresh-fruit samples, six per stage; coloured labels identify the three residual-range candidates. (b) Developmental versus nine-location log2 ranges for 11 name/formula overlaps. Points below y = x retain positive residual range excess. (c) Bounded envelope coverage C = min(Rdev/Rloc, 1), residual fraction max(1 - Rdev/Rloc, 0), validation tier and leave-one-location-out retention. (d) Monte Carlo sensitivity across Dirichlet stage-mixture α values and assumed lot-level drying log2 SD values; cells report observed location range divided by simulated q95. Gallic acid and ellagic acid are Tier 1; kaempferol 3-O-glucoside is Tier 2. The parameters are assumptions, fresh and dried samples are not matched, and no panel validates an origin marker.

Monte Carlo analysis exposed the dependence of that priority on unmeasured commercial-lot structure. With an assumed drying log2 SD of 0.25, gallic acid and ellagic acid exceeded the scenario q95 across all three Dirichlet mixture settings, whereas kaempferol 3-O-glucoside did so only at α = 2. With an assumed drying log2 SD of 0.5, no candidate exceeded q95 across all mixture settings. The simulation therefore quantifies robustness to explicit assumptions rather than replacing stage-controlled validation.

### 3.8 Reference-genome analysis prioritises biosynthetic gene sets

The species-specific reference genome identified 420 pathway records corresponding to 369 unique*R. chingii* genes. Seven visualised pathway groups contained 163 phenylpropanoid, 58 branched-chain-amino-acid, 55 terpenoid-backbone, 47 flavonoid, 42 alanine/aspartate/glutamate, 37 nitrogen-metabolism and 15 monoterpenoid genes. Counts are not additive because genes may occur in more than one KEGG pathway. The candidate screen included FLS, DFR, UGT, GDH and AST loci for sequence-level review (Supplementary Table S8).

Target-pathway genes were distributed across all seven linkage groups rather than confined to one region. Phenylpropanoid genes were most numerous on LG03, flavonoid genes on LG04 and monoterpenoid genes on LG07. None of the tested pathway-linkage-group combinations remained significant after false-discovery correction. These counts describe reference-genome capacity, not location-specific expression or chemical causality.

### 3.9 Sequence-resolved mapping recovers loci missed by KO screening

Four of six public biosynthetic genes met the high-confidence mapping rule (identity ≥90% and query coverage ≥95%).*RcFLS1* mapped to LG02.1317, *RcFLS2* to LG02.3010, *RcUGT75T4* to LG07.1014 and *RcUGT85A57* to LG03.1781. *RcDFR* mapped with 100.0% identity but only 82.4% query coverage, while *RcUGT75L20* mapped provisionally with 81.5% identity and 77.9% coverage. The short *RcDFR* alignment is therefore a model-curation flag rather than a high-confidence functional mapping. The two KO-missed UGT loci were LG07.1014 and LG03.1781 (Supplementary Table S8). Across the prioritised set, candidates LG07.4008 and LG07.4009 — adjacent to the characterised flavonoid UGT UGT72E2 in the UGT phylogeny and showing the highest template identities (58–67%) — also ranked among the top structural matches for kaempferol and quercetin, converging sequence-, tree- and structure-level evidence.

### 3.10 Whole-proteome UGT audit quantifies a KO annotation gap

PF00201 scanning identified 178 protein models in the*Rubus chingii* Hu v1.0 proteome. Of these, 130 had a hit ending within 60 amino acids of the protein C terminus, providing a simple completeness flag, whereas 48 were included as profile hits without that C-terminal positional support. Fifty-five of the 178 candidates (30.9%) carried a UGT-related KO label, one carried a discordant non-UGT label and 122 had no KO assignment. This conditional recall estimate is independent of the six-gene sequence-anchoring exercise and shows that family-level sequence evidence recovers a much larger candidate space than KO labels alone. The catalogue supports locus prioritisation, not a claim that every PF00201 hit is a functional flavonoid glycosyltransferase. Direct comparison with Shi et al. (2024) showed that all 172 published RchUGT loci were compiled in the current PF00201 catalogue, which also contained six additional models (Supplementary Table S8). Because the 172-member published catalogue was itself built by a family-profile search of the same genome, this complete recovery is reported as the expected consistency check it represents rather than an independent discovery. The six additional models are profile-defined candidates rather than newly validated flavonoid UGTs; their model lengths, PSPG-motif presence and C-terminal positional completeness are deposited with the Supplementary Data. Consistent with the observation that 48 of the 178 profile hits lack a C-terminal-positioned domain, these additions may include fragments or split gene models and are retained as operational family members pending transcript-supported curation.

#### 3.10.1 Species-matched controls quantify the sensitivity gained over KO-only screening

The PF00201 scan recovered all three public*R. chingii* UGT controls, whereas the prespecified KO-only flavonol-glycosyltransferase screen and the PF00201-plus-UGT-related-KO intersection each compiled none (Table 4). It should be noted that the prespecified KO-only screen was defined for flavonol glycosyltransferases, whereas the three controls are UGT75/UGT85 family members; their non-recovery in that screen therefore reflects the screen definition as much as the completeness of the KO annotation. The corresponding genome-wide candidate sets contained 178, 6 and 55 proteins, respectively. Across 10,000 random three-protein sets, the mean number of PF00201 hits was 0.0171 and the maximum was two hits (one set; 9830 sets had zero and 169 had one; no set had three; exact hypergeometric *P* = 1.53 × 10^−7^; plus-one empirical *P* = 9.999 × 10^−5^). Thus, family-profile screening increased sensitivity for this small positive-control set. The comparison does not establish that all PF00201 hits are functional UGTs or that family-profile screening has higher substrate-level precision. The empirical value is bounded by the 1/10,001 resolution of the finite randomisation, whereas the exact probability is not simulation-limited. This benchmark should be read as a sensitivity check rather than specificity evidence: because PF00201 covers only a small fraction of the proteome, the highly significant exact probability largely restates how few proteins the profile hits. A direct route-by-route comparison across independent family-detection methods (PSPG-motif search, InterPro and eggNOG annotation) would be more informative, and we note that such a comparison was not performed here. Under current database versions (PyHMMER 0.12.3), the PF00201 scan yields 189 models (179 at gathering cutoffs) with 58 carrying UGT-related KO labels, 2 discordant and 129 without; the K22794-only screen remains at 6 genes (Section 2.6.1).

**Table 4:** Species-matched UGT positive-control capture benchmark.

Screening strategy	Genome candidates	Public*R. chingii* UGT controls captured	Capture
KO-only flavonol-glycosyltransferase screen	6	0/3	
PF00201 family-profile scan	178	3/3	
PF00201 plus UGT-related KO	55	0/3	

Neither of the two near-identical UGT loci was compiled by the prespecified KEGG-orthology candidate screen. Thus, sequence-level anchoring expanded the species-specific candidate set beyond KO-only annotation. The*RcDFR* reference model was shorter than the public coding sequence, whereas *RcUGT75T4* was embedded in an unusually long reference model, consistent with possible gene-model truncation or fusion. These structural flags require transcript-supported curation and were not interpreted as functional differences.

### 3.11 TPS candidates provide a genome-level context for volatile priorities

The Pfam scan detected 67 protein models containing at least one TPS domain. Forty-nine models contained both PF01397 and PF03936, 15 contained only the C-terminal domain and three contained only the N-terminal domain. Thirty-two models combined a C-terminal domain with a class-I or class-II catalytic motif and formed the motif-supported candidate set. This hierarchy preserves possible fragments for curation without treating them as equivalent to catalytically plausible full models.

The motif-supported candidates were non-randomly distributed across the reference genome. LG03 contained 17 of 32 candidates, compared with 5.1 expected from its share of all predicted proteins, corresponding to 3.3-fold enrichment (hypergeometric*P* = 1.52 × 10^−6^; BH *q* = 1.21 × 10^−5^). The remaining candidates occurred on LG01 (4), LG02 (5), LG05 (2) and LG06 (4), with none on LG04 or LG07. Within LG03, the median adjacent physical distance was 54.8 kb, compared with 1.41 Mb across 10,000 random 17-gene sets (plus-one *P* = 9.999 × 10^−5^). This supports physical co-localisation, not tandem duplication or shared-function inference. The difference between the LG07 pathway count and the LG03 motif-supported TPS count shows why pathway-level counts cannot substitute for family-resolved discovery.

Only 25 of the 32 motif-supported candidates had a TPS-specific KO assignment. Six had no KO and one carried a discordant non-TPS annotation, giving a TPS-specific KO recall of 78.1% and an audit-gap fraction of 21.9%. LG03.2235 encoded a 1234-aa model with both TPS domains and catalytic motifs but carried an MRE11 DNA-repair KO. LG02.1599 was also unusually long, although its ent-copalyl diphosphate synthase assignment was pathway-consistent. Both models were taken forward for coordinate and transcript review.

### 3.12 Comparative genomes resolve asymmetric model-boundary priorities

Of the 67 TPS-domain protein models, 66 mapped to official GFF3 features. LG03.2235 contained four nested models within a long intron without exon overlap, whereas LG02.1599 had a weaker opposite-strand intronic overlap. In*r. idaeus*, the LG03.2235 N-terminal and TPS segments occupied two non-overlapping, same-strand homologous blocks separated by 4.5 kb in the flanking-gene interval. In *R. occidentalis* v3.0, the same segments mapped at 95.2% and 90.5% identity to two separate proteins, Ro03_G18118 and Ro03_G32701, separated by 46.7 kb. The TPS segment and nested *R. chingii* model LG03.2237 shared Ro03_G32701 as their best black-raspberry match. These two independent comparisons support a fused-model annotation concern for LG03.2235.

The transcript evidence was concordant with, but not decisive for, the comparative result. LG03.2235 had ordinary support for 21 of 26 annotated junctions, yet the 22,832-bp composite boundary had zero exact junction reads in all 12 fruit libraries. By contrast, both LG02.1599 segments mapped to one*R. occidentalis* protein and one continuous *R. idaeus* homologous interval; it therefore remains a lower-priority transcript-boundary review rather than a comparative split-model candidate. Public long reads consisted of genomic WGS runs, not Iso-Seq or other transcript long reads, and cannot resolve the splice model. Comparative evidence supports re-curation of LG03.2235 but does not itself define the corrected exon structure or prove a biological fusion transcript.

Mapping volatile features to characterised reference branches provided a bounded interpretation of the TPS phylogeny (Fig. 6). Linalool was associated with TPS-g or TPS-b/g reference context, whereas α-terpineol and limonene were associated with TPS-b monoterpene context. Benzaldehyde and the 2-heptenal feature were excluded from TPS linkage because they are aromatic- and fatty-acid-derived aldehydes, respectively. These mappings nominate branches for future testing; they do not assign an enzyme product to an *R. chingii* locus.

**Fig. 6:**
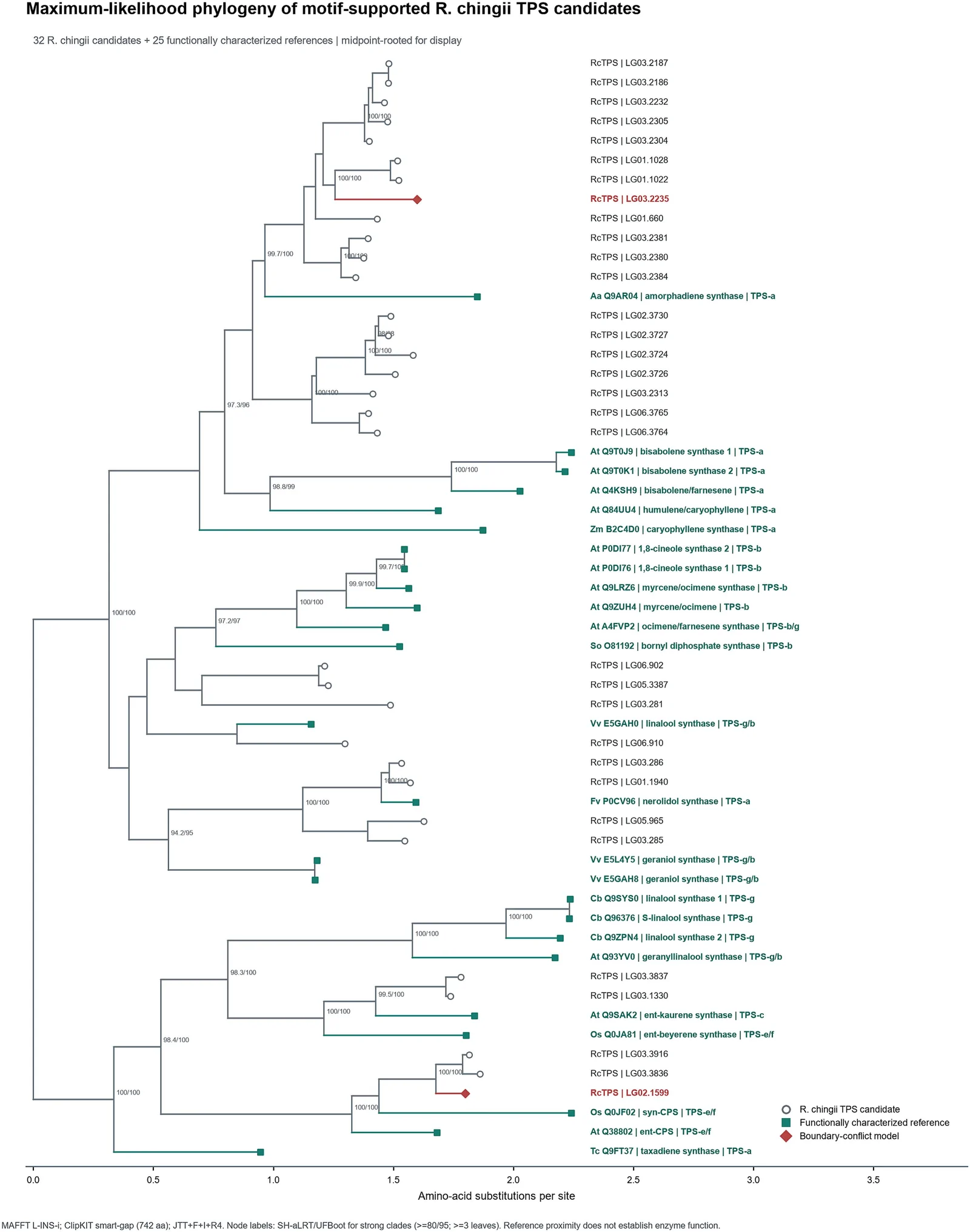
Maximum-likelihood phylogeny of 32 motif-supported *R. chingii* TPS candidates and 25 functionally characterised reference proteins. Candidate proteins are shown as open circles, characterised references as green squares and the two long boundary-priority models as red diamonds. Reference labels report species, accession, verified product and broad TPS context. Branch proximity defines test priority only and does not establish an *R. chingii* enzyme product. The reference set is functionally characterised but not Rosaceae-enriched; candidate placement is therefore interpreted at branch level only, and the alignment and tree files are deposited in the Supplementary Data. (For interpretation of the references to colour in this figure legend, the reader is referred to the web version of this article.)

## 4 Discussion

### 4.1 Two sequential gates convert uncertainty into validation priorities

This study addresses a practical authenticity problem: commercial dried fruits can be grouped by producing location, but harvest maturity is often unknown. The first evidence gate asks whether a location range exceeds the complete observed developmental envelope before a chemical candidate is promoted. The second asks whether chemistry-linked UGT/TPS loci survive species-specific profile, motif, transcript and comparative-genome review. This two-gate design converts two common uncertainties, missing maturity metadata and incomplete functional labels, into auditable validation priorities while preserving the distinction between nomination and validation (Fig. 7a).

**Fig. 7:**
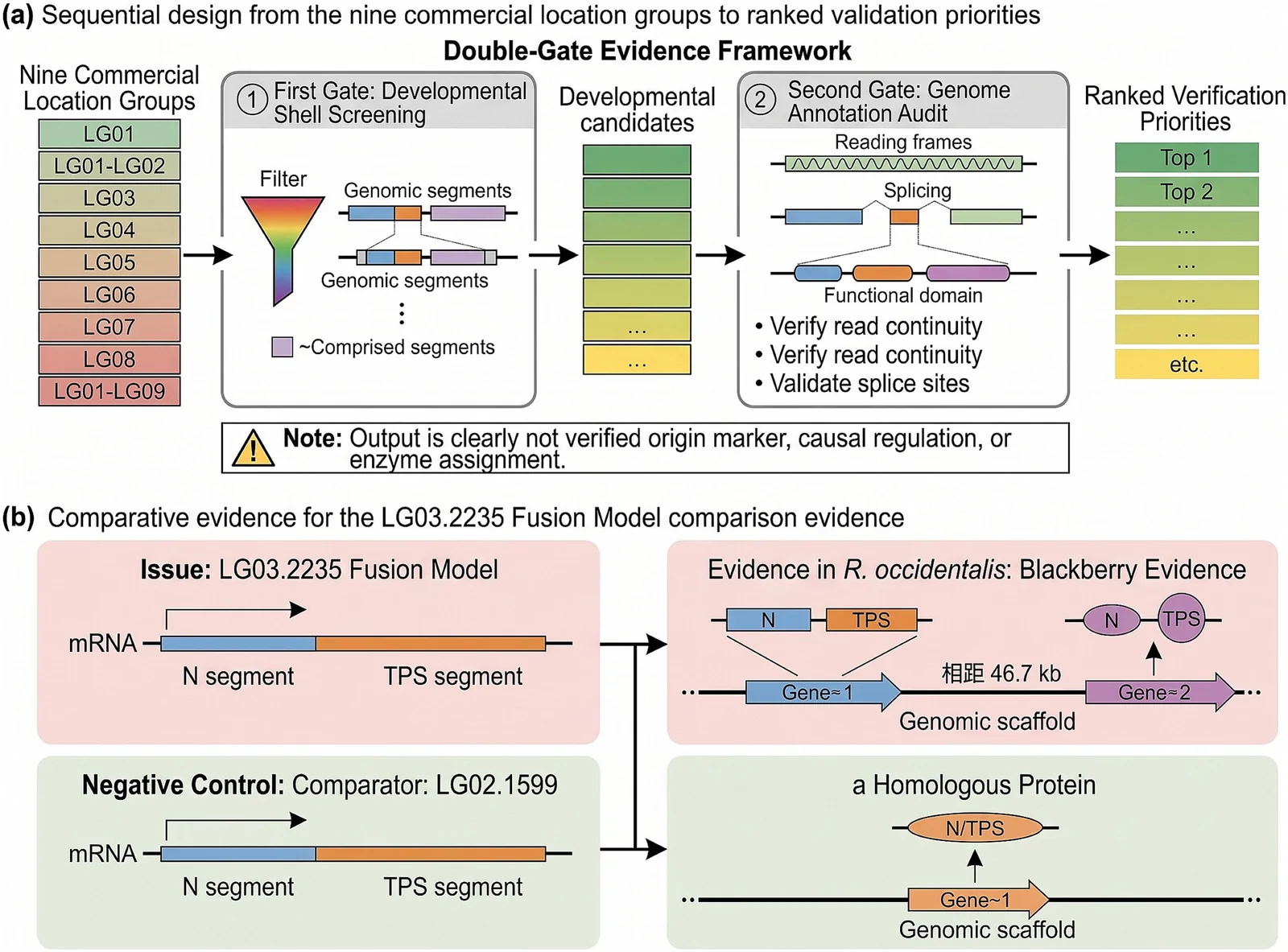
Two-gate evidence framework and comparative boundary-audit summary. (a) Sequential design from the nine commercial location groups through developmental-envelope screening (Gate 1) and genome-annotation audit (Gate 2) to ranked validation priorities; the output is explicitly not a validated origin marker, causal regulation or enzyme assignment. (b) Comparative evidence for the LG03.2235 fused-model concern: the N and TPS segments map to two separate proteins in *R. occidentalis* (46.7 kb apart), in contrast to the negative comparator LG02.1599, whose segments map to a single homologous protein.

Developmental-envelope coverage does not estimate a causal percentage.*C* = 1 means only that the observed four-stage range was at least as large as the location range; it does not prove that development produced the location ranking. Nevertheless, the residual-range analysis is not a purely negative filter. Gallic acid and ellagic acid met both leave-one-location-out and moderate-scenario criteria and therefore form Tier 1; kaempferol 3-O-glucoside forms Tier 2 because its scenario robustness was weaker. These tiers determine testing order, not marker validity.

The Monte Carlo grid makes the assumptions visible. Priority changed with the concentration of stage mixtures and with hypothetical drying variability, and no feature was robust across every high-uncertainty setting. Fresh-fruit developmental measurements and dried commercial-fruit profiles also come from different samples and analytical contexts. The simulation is therefore a sensitivity analysis, not a reconstructed harvest history or a substitute for measured drying factors.

Chemical-identity evidence remains an independent requirement within the first gate. The LC-MS/MS overlaps are putative unless authentic-standard support is explicit, while the volatile profiles rely on library/retention-index evidence without a complete RI ledger. The developmental-envelope tier determines validation order; it cannot upgrade chemical identity. This separation is especially important for the discordant kaempferol KEGG label and for commercial processing effects documented in berry and food systems (Teegarden et al., 2019; Toydemir et al., 2022).

The genome analysis adds a second methodological result. Family-profile screening recovered UGT and TPS candidates that KO-only annotation missed, while the four-stage expression-metabolite audit showed that*n* = 4 stage means cannot support multiplicity-resolved regulatory links. Reporting that negative resolution limit prevents phylogenetic proximity or shared developmental direction from being misread as pathway regulation.

Comparative evidence changes the status of one focal model. LG03.2235 combines an MRE11-like N segment and TPS C segment, lacks reads across its decisive boundary and resolves into separate homologous blocks or proteins in two near relatives. The agreement among annotation conflict, transcript non-support and comparative separation supports a specific fused-model annotation concern (Fig. 7b). LG02.1599 supplies an internal negative comparator because both near relatives retain one homologous model. On the flavonoid axis, the sequence-resolved FLS/DFR priorities are consistent with the pathway competition reported by Lei et al. (2023), but the present data do not establish flux or regulation. Even for LG03.2235, the corrected transcript must still be established by transcript long reads or targeted splice-aware curation.

The amino-acid profiles add a nutritional composition axis to the volatile and flavonoid measurements. Glutamate and aspartate dominate the acid-hydrolysate pool, consistent with the central role of glutamate metabolism in plants (Forde & Lea, 2007), but amino-acid chemometrics can support authenticity only when free-amino-acid methods, moisture basis and independent validation are documented (Wistaff et al., 2021). Universal tryptophan non-detection remains method-limited because prolonged acid hydrolysis compromises its recovery.

### 4.2 Sensory, nutritional and maturity relevance of candidate chemistry

The candidate library has direct food-chemistry relevance while remaining provisional. Linalool, α-terpineol and limonene represent aroma-relevant volatile classes; glutamate- and aspartate-related pools describe composition rather than sensory intensity; and rutin-, gallic-acid-, kaempferol- and ellagic-acid-related features are phenolic priorities rather than confirmed bioactive or authenticity endpoints. This nomination-versus-validation distinction follows the quality-marker (Q-marker) principle that candidate markers require systematic validation before standard-setting use (Liu et al., 2016; Ren et al., 2020; Yang et al., 2017). Developmental-envelope screening separates eight development-sensitive descriptors from two Tier 1 and one Tier 2 validation candidates.

### 4.3 Family-aware annotation links food chemistry to genomic candidates

The UGT and TPS analyses provide two independent demonstrations of the same annotation problem. For UGTs, PF00201 recovered all three species-matched positive controls while the KO-only screen compiled none, and only 55 of 178 family-profile candidates carried UGT-related KO labels. For TPSs, seven motif-supported candidates lacked a TPS-specific KO and one carried an unrelated DNA-repair annotation. Random-set benchmarking shows that three-of-three UGT recovery is highly unlikely under arbitrary proteome sampling, but it does not measure specificity. Together, these results support a tiered audit in which KO labels provide pathway context, family profiles maximize candidate recovery, and sequence/coordinate evidence identifies models requiring curation.

The LG03 concentration offers a candidate genomic context for terpene-synthase expansion, but the model audit is more specific than a chromosome-level count. Linalool, α-terpineol and limonene motivate TPS-g/TPS-b branch-level tests, given the ecological and aroma functions of terpene natural products (Gershenzon & Dudareva, 2007), whereas benzaldehyde and 2-heptenal are not TPS products. On the flavonoid side, the phenylpropanoid pathway provides the candidate biosynthetic context for the FLS/DFR/UGT audit (Fraser & Chapple, 2011). For LG03.2235, two near-relative comparisons support separation of the MRE11-like and TPS components. This result prioritises reference annotation repair; it does not identify the TPS product or connect genome variation to the commercial location rankings.

### 4.4 Transferable use in future food-origin studies

The practical output is a developmental-envelope-ranked chemical library, a reproducible scenario-analysis workflow and a comparative gene-model audit, not a validated origin classifier. In a future commercial-fruit validation, candidates should first pass authentic-standard confirmation and moisture-aware quantification, then be tested in independent lots with recorded maturity, drying and storage. Only after those gates should location-grouped classification or locus-specific expression be considered; any such classifier must fit every preprocessing and feature-selection step inside location-grouped training folds and report per-location held-out predictions, calibration and uncertainty (Varma & Simon, 2006). The workflow can be transferred to other processed fruits in which harvest stage is missing or incompletely recorded.

### 4.5 Candidate prioritisation and validation for future food-origin research

The tiered screen defines an economical validation sequence. Gallic acid and ellagic acid were more robust than kaempferol 3-O-glucoside under moderate assumed drying variability, but that ordering remains scenario-dependent. Targeted standard confirmation and stage-controlled commercial-lot replication should therefore begin with Tier 1, followed by Tier 2. The remaining eight overlaps remain useful development-sensitive quality descriptors rather than discarded features.

The fresh-to-dried transfer remains the principal chemical limitation. Drying may amplify, attenuate or reorder stage trajectories, and no empirical drying factor was available. By showing results across three assumed log2 drying SDs rather than selecting one convenient value, the analysis makes this uncertainty reviewable. The developmental envelope and validation tiers should be updated when paired fresh/dried measurements become available.

The public transcriptome supplies expression and splice context, but the exact four-stage permutation audit demonstrates why apparent trajectory concordance cannot be promoted to regulation. No gene-metabolite pair survived BH correction, and public long reads were genomic rather than transcriptomic. The strongest genome conclusion therefore rests on cross-species model separation plus junction non-support, not on an unsupported expression-metabolite mechanism.

## 5 Conclusion

This study applies two sequential evidence gates to commercial*R. chingii* fruit. The observed developmental envelope covered the location range for eight of 11 overlapping metabolites; gallic acid and ellagic acid met Tier 1 robustness criteria, whereas kaempferol 3-O-glucoside met Tier 2 criteria. The genome-annotation gate recovered all 172 published RchUGTs, identified six additional profile-defined UGT candidates and placed 32 motif-supported TPS candidates in sequence-resolved context. Two near-relative genomes independently supported re-curation of LG03.2235 while leaving LG02.1599 as a negative comparator. The framework yields testable chemical and genomic priorities without presenting them as validated origin markers, causal regulation or enzyme assignments.

## Declaration of generative AI and AI-assisted technologies

Generative AI was used to assist with language editing and structural review during manuscript revision. The authors verified the scientific content, included responsibility for all claims, and did not use AI to generate or alter experimental data.

## CRediT authorship contribution statement

**Wenkai Duan:** Writing – original draft, Resources, Formal analysis, Data curation, Conceptualization. **Zhenxun Wei:** Resources, Methodology. **Wenli Gao:** Software, Project administration. **Lingyao Peng:** Writing – review & editing, Writing – original draft, Resources, Data curation, Conceptualization.

## Code availability

All scripts used to generate the public-data evidence tables, UGT/TPS audits, developmental-envelope analysis, comparative boundary audit, figures and manuscript are included in the accompanying Supplementary Data and Code archive together with package versions, a file manifest and SHA-256 checksums.

## Funding

This research was supported by the Zhejiang Medical and Health Science and Technology Plan Project (Grant No. 2025HY0730).

## Declaration of competing interest

The authors declare that they have no known competing financial interests or personal relationships that could have appeared to influence the work reported in this paper.

## Data Availability

The public developmental RNA-seq data are available from the NCBI Sequence Read Archive under BioProject PRJNA671545. The *Rubus chingii* Hu v1.0 assembly and annotation (Genome Database for Rosaceae Analysis 11,326,199, publication dataset tfGDR1051; raw reads NCBI BioProject PRJNA666516; Wang et al., 2021) and the *R. occidentalis* whole-genome assembly v3.0 (VanBuren et al., 2018) were obtained from the Genome Database for Rosaceae, and the *R. idaeus* comparison used GenBank assembly GCA_047496405.1. The publication-associated developmental metabolite table was obtained from the supplementary record of Chen et al. (2021). The processed chemical matrices, amino-acid source matrix, UGT/TPS catalogues, full RNA-seq count and expression tables, exact junction audit, developmental-envelope scores, robustness outputs, comparative-genome tables, figure source data and analysis scripts are provided in the accompanying Supplementary Data and Code archive submitted with this article. No public DOI is claimed until the archive is deposited in a repository.
