# Supplementary material for: Developmental-envelope screening and genome-annotation audit prioritize chemical authenticity candidates in commercial Rubus chingii fruit
Source: Food Chem X. 2026 Oct 16;40:104635. doi: 10.1016/j.fochx.2026.104635 (PMC13697275; doi:10.1016/j.fochx.2026.104635)
Supplement: Supplementary file 1 [file mmc1.docx]

Supplementary Material

Developmental-envelope screening and genome-annotation audit prioritize chemical authenticity candidates in commercial Rubus chingii fruit

# Supplementary Methods

## S1.1 HS-SPME-GC-MS instrument parameters

After 10 min preheating at 60 °C, headspace extraction used a 50/30-µm DVB/CAR/PDMS fibre (Sigma) for 20 min at 60 °C, desorption at 250 °C for 5 min; the fibre was baked for 5 min between injections, and new fibres were conditioned for 2 h before first use. GC-MS: DB-5MS column (30 m × 0.25 mm × 0.25 µm); splitless injection at 250 °C (3.5-min solvent delay); oven 40 °C (5 min) to 280 °C at 6 °C/min (5-min hold); helium 1.0 mL/min; EI 70 eV (source 230 °C, quadrupole 150 °C, transfer line 280 °C); m/z 50–500.

## S1.2 Amino-acid HPLC conditions

Mobile phase A: 4.0 g sodium acetate, 0.3 mL triethylamine and 10 mL tetrahydrofuran per litre of water (pH 7.2); mobile phase B: methanol/acetonitrile/water (45:45:10, v/v/v). Column Diamonsil-plus C18-A (4.6 × 250 mm, 5 µm) at 40 °C over 43 min; detection at 338 nm (8–25 min) and 262 nm (27–43 min).

## S1.3 HPLC-UV flavonoid gradient

Methanol (A) / 0.1% formic acid in water (B): 30–40% A (0–10 min), 40–60% A (10–20 min), 60–80% A (20–30 min), 80–30% A (30–35 min).

## S1.4 LC-MS/MS (widely targeted flavonoid profiling) conditions

UPLC: Agilent SB-C18 (1.8 µm, 2.1 × 100 mm); mobile phase water (A) and acetonitrile (B), each with 0.1% formic acid; gradient 95% A/5% B to 5% A/95% B over 0–9 min, held 1 min, returned to 95% A/5% B within 1.10 min and held for 2.9 min; flow 0.35 mL/min; column oven 40 °C; injection 4 µL. MS: AB 4500 QTRAP with ESI Turbo Ion-Spray (550 °C; IS 5500 V positive / −4500 V negative; GSI/GSII/CUR 50/60/25 psi); MRM with scheduled transitions; Analyst 1.6.3 acquisition and MultiQuant processing against the MWDB database.

## S1.5 RNA-seq processing

FASTQ files verified against ENA-reported byte counts; FastQC quality assessment; Trimmomatic trimming (LEADING:3 TRAILING:3 SLIDINGWINDOW:4:15 MINLEN:36); alignment to the Rubus chingii Hu v1.0 reference with Subjunc 2.0.2 (paired-end, splice-aware); quantification with featureCounts 2.0.3 (-p --countReadPairs -B -C -T 20 -J -G), counting concordant paired fragments and excluding orphan/chimeric pairs; DESeq2 stage contrasts adjusted over the full transcriptome; TPM generated for visualisation.

## S1.6 UGT structural modelling and docking

Thirty prioritised candidates were modelled as C-terminal catalytic domains (350 aa) with ESMFold against eight plant UGT crystal structure templates (phmmer screen; 124 candidates exceeded 35% template identity). Docking used AutoDock Vina with the search grid centred on the PSPG-box tryptophan; ligands: kaempferol, quercetin, gallic acid and ellagic acid. Docking scores are structural-compatibility rankings, not evidence of catalytic activity. Full rankings and model quality metrics are deposited with the Supplementary Data and code archive.

## S1.7 TPS candidate definitions, thresholds and coordinate rules

The motif-supported TPS set required a C-terminal TPS domain (Pfam PF03936) together with a class-I or class-II catalytic signature; domain assignments follow Pfam gathering thresholds. GFF3 coordinate audit: TPS candidates were mapped to gene, transcript, exon and CDS records; coordinate overlap was treated as a model-boundary flag rather than evidence of tandem duplication. Candidate-level records are deposited with the Supplementary Data.

## S1.8 Standalone detection-route pipeline

The dedicated PSPG-profile, InterPro and eggNOG comparison (Section 2.6.1) is deposited with the code archive for local completion.

# Supplementary Tables

# Table S1. Candidate inventory (18 non-redundant chemical entities after synonym resolution)

| Entity | Evidence source | Envelope outcome |
| --- | --- | --- |
| Gallic acid | LC-MS/MS (putative) | Tier 1 |
| Ellagic acid | LC-MS/MS (putative) | Tier 1 |
| Kaempferol 3-O-glucoside | LC-MS/MS (putative) | Tier 2 |
| Naringenin chalcone | LC-MS/MS (putative) | Development-sensitive |
| Naringenin | LC-MS/MS (putative) | Development-sensitive |
| Quercetin | LC-MS/MS (putative) | Development-sensitive |
| Rutin (= quercetin-3-O-rutinoside) | LC-MS/MS (putative) | Development-sensitive |
| Gallotannin | LC-MS/MS (putative) | Development-sensitive |
| Dihydroquercetin | LC-MS/MS (putative) | Development-sensitive |
| Dihydrokaempferol | LC-MS/MS (putative) | Development-sensitive |
| Kaempferol | LC-MS/MS (putative) | Development-sensitive |
| Linalool | GC-MS/RI (putative) | Aroma priority |
| Benzaldehyde | GC-MS/RI (putative) | Aroma priority |
| 2-Heptenal isomer (stereochemistry unresolved) | GC-MS/RI (putative) | Aroma priority |
| α-Terpineol | GC-MS/RI (putative) | Aroma priority |
| Limonene | GC-MS/RI (putative) | Aroma priority |
| Glutamate pool (Glu+Gln) | Amino-acid reports (Table S3) | Composition priority |
| Aspartate pool (Asp+Asn) | Amino-acid reports (Table S3) | Composition priority |

# Table S2. Requirements for a reproducible future origin-classification evaluation

| Component | Requirement |
| --- | --- |
| Sample matrix | Independently sampled material with recorded maturity, drying and storage; sample-level observations preserved |
| Grouped resampling | All samples from one location kept in the same fold |
| Feature selection | Documented OPLS-DA or alternative; every preprocessing and selection step fitted inside training folds only (Varma & Simon, 2006) |
| Held-out predictions | Per-location predictions reported with calibration and uncertainty |
| Chemical confirmation | Authentic-standard confirmation and moisture-aware quantification before modelling |

# Table S3. Amino-acid source matrix (mg/kg as-submitted dried material, from the nine laboratory reports ZX210624-240101–240109)

| Analyte (报告项目 / abbreviation) | F1 亳州(BZ) | F2 院桥(YQ) | F3 缙云(JY) | F4 嵊县(SX) | F5 三门(SM) | F6 广德(GD) | F7 永嘉(YJ) | F8 磐安栽培(PA) | F9 磐安野生(PAYS) |
| --- | --- | --- | --- | --- | --- | --- | --- | --- | --- |
| 天冬氨酸 / Asp (Asp+Asn) | 12,347.7 | 11,298.9 | 11,987.2 | 9,353.2 | 8,430.1 | 7,140.8 | 10,948.2 | 9,491.4 | 6,891.9 |
| 苏氨酸 / Thr | 3,458.8 | 3,760.7 | 3,213.4 | 3,692.3 | 3,243.1 | 3,293.8 | 3,446.7 | 3,778.2 | 2,948.6 |
| 丝氨酸 / Ser | 3,370.9 | 3,978.7 | 3,434.2 | 3,895.2 | 3,366.0 | 3,666.6 | 3,873.2 | 4,076.4 | 3,171.4 |
| 谷氨酸 / Glu (Glu+Gln) | 10,412.1 | 10,424.0 | 9,936.9 | 10,680.6 | 9,450.0 | 9,731.2 | 11,984.1 | 10,734.9 | 9,680.8 |
| 脯氨酸 / Pro | 3,842.8 | 4,243.0 | 3,967.7 | 3,718.7 | 3,492.0 | 3,104.3 | 3,558.0 | 4,457.1 | 2,754.2 |
| 甘氨酸 / Gly | 3,842.3 | 4,070.8 | 3,617.8 | 3,996.7 | 3,865.5 | 3,845.3 | 4,123.6 | 4,260.9 | 3,383.1 |
| 丙氨酸 / Ala | 3,838.1 | 4,436.0 | 3,777.7 | 4,228.6 | 3,833.1 | 3,597.6 | 3,855.1 | 4,209.9 | 3,313.2 |
| 胱氨酸 / Cys | 704.9 | 811.6 | 726.5 | 741.0 | 767.1 | 774.8 | 597.6 | 776.5 | 653.0 |
| 缬氨酸 / Val | 4,064.0 | 4,466.9 | 3,724.1 | 4,374.2 | 3,980.5 | 3,557.4 | 3,724.3 | 4,465.0 | 3,529.3 |
| 蛋氨酸 / Met (reported as 甲硫氨酸) | 1,659.8 | 1,882.0 | 1,591.2 | 1,634.6 | 1,445.8 | 1,457.9 | 1,338.3 | 1,782.4 | 1,460.0 |
| 异亮氨酸 / Ile | 3,705.7 | 4,167.4 | 3,449.0 | 4,104.5 | 3,775.7 | 3,353.0 | 3,476.1 | 4,013.0 | 3,483.8 |
| 亮氨酸 / Leu | 5,493.5 | 6,521.7 | 5,376.8 | 6,367.8 | 5,856.5 | 5,491.4 | 5,425.5 | 6,113.9 | 5,043.1 |
| 酪氨酸 / Tyr | 3,704.1 | 4,162.7 | 3,124.9 | 3,694.7 | 3,441.0 | 3,154.8 | 2,944.7 | 4,002.2 | 3,048.4 |
| 苯丙氨酸 / Phe | 3,865.8 | 4,300.8 | 3,428.2 | 4,270.2 | 3,825.3 | 3,490.4 | 3,398.5 | 4,243.2 | 3,389.3 |
| 赖氨酸 / Lys | 4,035.2 | 4,615.7 | 3,872.0 | 4,497.9 | 4,033.6 | 3,649.1 | 3,410.5 | 4,357.1 | 3,148.0 |
| 组氨酸 / His | 3,230.9 | 3,671.9 | 2,981.7 | 3,388.2 | 3,356.6 | 3,239.2 | 2,938.9 | 3,575.4 | 2,795.4 |
| 精氨酸 / Arg | 6,356.5 | 6,725.3 | 5,720.3 | 6,281.1 | 5,815.5 | 5,393.6 | 7,423.6 | 6,635.8 | 5,057.7 |
| Σ 17 quantified analytes | 77,933.1 | 83,537.9 | 73,929.5 | 78,919.5 | 71,977.5 | 67,941.1 | 76,466.9 | 80,973.1 | 63,751.2 |

Tryptophan not detected in any report (excluded). Glutamate and aspartate are combined Glu+Gln and Asp+Asn pools after acid hydrolysis. Methionine is reported as 甲硫氨酸 in the Chinese reports. Moisture correction was not documented. Values are displayed to one decimal place; the Σ row is computed from unrounded report values, so re-summing the displayed columns may differ by ≤0.2 mg/kg.

# Table S4. Amino-acid compositional groupings by location (shares of the 17-analyte total, %)

| Location | Glu+Asp (umami) | Sweet-5 (Gly/Ala/Ser/Thr/Pro) | Bitter-7 (Val/Met/Leu/Ile/Phe/His/Arg) |
| --- | --- | --- | --- |
| F1 亳州(BZ) | 29.2 | 23.5 | 36.4 |
| F2 院桥(YQ) | 26.0 | 24.5 | 38.0 |
| F3 缙云(JY) | 29.7 | 24.4 | 35.5 |
| F4 嵊县(SX) | 25.4 | 24.7 | 38.5 |
| F5 三门(SM) | 24.8 | 24.7 | 39.0 |
| F6 广德(GD) | 24.8 | 25.8 | 38.2 |
| F7 永嘉(YJ) | 30.0 | 24.7 | 36.3 |
| F8 磐安栽培(PA) | 25.0 | 25.7 | 38.1 |
| F9 磐安野生(PAYS) | 26.0 | 24.4 | 38.8 |

Compositional groupings, not sensory intensities. Location ranges: Glu+Asp 24.8–30.0%; Sweet-5 23.5–25.8%; Bitter-7 35.5–39.0% (Section 3.1.2).

# Table S5. Independent-unit ledger

| Evidence layer | Independent unit | Replication status | Permitted use | Inference boundary |
| --- | --- | --- | --- | --- |
| Volatile HS-SPME-GC-MS table | Location group (n = 9 summaries) | Compiled; biological replicates not preserved | Descriptive location profiles; candidate prioritisation | No SD/ANOVA/q from summary columns; putative library/RI annotation |
| LC-MS/MS feature table (318 rows) | Location group (n = 9 summaries) | Compiled; three pooled-QC columns | Descriptive rankings; targeted confirmation priority | Identity putative without authentic standards; no classifier |
| Amino-acid laboratory reports | One submitted profile per location | Single submitted profile; moisture basis undocumented | Compositional description (Tables S3–S4) | No biological-replicate error bars or significance tests |
| Electronic-nose export | Technical repeat (4 measurements/group) | Technical, not biological | Measurement-repeatability assessment (CV, PCA) | No geographic classifier or origin inference |
| Electronic-tongue report | Report-derived outputs only | Response matrix unrecoverable | Descriptive instrument summary | Not independent geographic evidence; report-level record only (Fig. S6) |
| Public developmental metabolome | Fruit stage (4 stages × 6 samples) | Independent public dataset | Developmental-envelope context (Gate 1) | Not pooled with commercial dried-fruit data |
| Public developmental RNA-seq (PRJNA671545) | Stage library (12 libraries) | Independent public dataset | Expression and splice-junction context | n = 4 stage means: minimum exact two-sided P = 0.0833; no regulation inference |
| Reference genomes (R. chingii v1.0; R. idaeus; R. occidentalis) | Genome-wide catalogue | Annotation resource | Locus prioritisation; model-boundary audit (Gate 2) | No expression, activity or product assignment inferred |

# Table S6. Developmental-envelope scores for the 11 cross-dataset overlaps (log2 units)

| Overlap | Rloc (9-location range) | Rdev (4-stage range) | E (residual excess) | Stage effect (BH q) | LOO retention | Tier |
| --- | --- | --- | --- | --- | --- | --- |
| Gallic acid | 3.4 | 3.0 | 0.41 | q = 0.00522 | 8/9 | Tier 1 |
| Kaempferol 3-O-glucoside | 1.3 | 0.92 | 0.38 | q = 0.00325 | 9/9 | Tier 2 |
| Ellagic acid | 1.49 | 0.57 | 0.92 | q = 0.0143 | 9/9 | Tier 1 |
| Naringenin chalcone | 1.36 | 2.16 | 0 | q = 0.00058 | — | Development-sensitive |
| Naringenin | 1.99 | 2.16 | 0 | q = 0.00058 | — | Development-sensitive |
| Quercetin | 1.15 | 2.01 | 0 | q = 0.000997 | — | Development-sensitive |
| Rutin | 0.95 | 1.54 | 0 | q = 0.000997 | — | Development-sensitive |
| Gallotannin | 1.15 | 1.49 | 0 | q = 0.0438 | — | Development-sensitive |
| Dihydroquercetin | 0.69 | 1.4 | 0 | q = 0.000708 | — | Development-sensitive |
| Dihydrokaempferol | 1.01 | 1.29 | 0 | q = 0.00129 | — | Development-sensitive |
| Kaempferol | 1.18 | 1.27 | 0 | q = 0.000997 | — | Development-sensitive |

Monte Carlo scenario grid (Dirichlet α = 0.5/1/2 × drying log2 SD = 0/0.25/0.5; 30,000 runs per setting): gallic acid and ellagic acid exceeded the simulated q95 in all three moderate-drying settings; kaempferol 3-O-glucoside in 1/3 (α = 2 only); at drying SD = 0.5 no candidate exceeded q95 across all mixture settings.

# Table S7. Expression–metabolite coupling audit and comparative-boundary summary

| Item | Result |
| --- | --- |
| RNA-seq quantification | 31,994 annotated models; per-library mapping 96.2–97.0%; 69.5–78.3% of fragments assigned |
| Candidate catalogue | 247 unique TPS/UGT and sequence-anchored candidates; 152 with estimable stage contrasts; 104 BH-significant in ≥1 contrast (79/110 estimable UGT; 23/40 estimable TPS) |
| Coupling audit design | 147 expressed, non-constant candidate genes × 21 metabolites = 3,087 pairs; exact two-sided P over all 24 stage permutations; BH-adjusted |
| Coupling audit outcome | Minimum attainable exact P = 0.0833; minimum q = 0.411; no pair passed BH correction — trajectories are parallel developmental priorities, not regulation |
| Long-model junction audit | LG02.1599: 13/16 junctions supported (1,114 exact reads); LG03.2235: 21/26 supported (1,382 reads); both composite boundary junctions with zero reads in all 12 libraries; longest annotated introns 871 bp (LG02.1599) and 22,832 bp (LG03.2235) |
| Comparative boundary evidence | LG03.2235 N/TPS segments occupy two non-overlapping homologous blocks in R. idaeus (4.5 kb apart) and two separate R. occidentalis proteins (95.2%/90.5% identity, 46.7 kb apart); LG02.1599 segments map to one homologous protein/interval in both relatives (negative comparator) |

# Table S8. Public biosynthetic sequences and reference set used for sequence anchoring and phylogeny

| Item | Mapping / role | Confidence / note |
| --- | --- | --- |
| RcFLS1 | LG02.1317 | High confidence (≥90% identity, ≥95% coverage) Source: Lei et al. (2023) |
| RcFLS2 | LG02.3010 | High confidence Source: Lei et al. (2023) |
| RcDFR | Reference-model shorter than public CDS | 100.0% identity, 82.4% coverage — model-curation flag Source: Lei et al. (2023) |
| RcUGT75L20 | Provisional mapping | 81.5% identity, 77.9% coverage Source: Huang et al. (2024) |
| RcUGT75T4 | LG07.1014 | High confidence; missed by the KO-only screen Source: Huang et al. (2024) |
| RcUGT85A57 | LG03.1781 | High confidence; missed by the KO-only screen; obligatory inclusion in modelling set Source: Huang et al. (2024) |
| RchUGT catalogue comparison (Shi et al., 2024) | 172/172 published loci recovered in the PF00201 catalogue; 6 additional profile-defined models | Expected consistency check (same family-profile logic); additions retained as operational members pending transcript-supported curation |
| 25 characterised TPS reference proteins | Functional reference set for the TPS phylogeny (Fig. 6) | Curated UniProt records; alignment and tree files deposited with the Supplementary Data |
| 32 per-candidate TPS subfamily assignments | Branch placement of the motif-supported candidates | Deposited with the alignment/tree files |

# Supplementary Figures (captions)

**Supplementary Fig. S1.** UGT phylogeny of the 30 docking-prioritised candidates (blue circles) among 100 characterised plant UGT references (grey squares). Maximum-likelihood tree (IQ-TREE 2.4.0, LG+G) from the MAFFT alignment of full-length proteins; branch lengths are substitutions per site. Placement is used for functional-context annotation only.

**Supplementary Fig. S2.** Structural compatibility ranking of the 30 prioritised UGT candidates. Vina scores (kcal/mol) for the four aglycone acceptors docked with the grid centred on the PSPG-box tryptophan; candidates ordered by mean aglycone score. Red labels mark the low-confidence model LG03.3408 (mean pLDDT 58), excluded from the ranking. Scores are structural-compatibility rankings, not evidence of catalytic activity.

**Supplementary Fig. S3.** High-resolution rendering of the main-text Fig. 6 maximum-likelihood phylogeny of the 32 motif-supported Rubus chingii TPS candidates and 25 functionally characterised reference proteins, with legible UFBoot2 branch-support values and a scale bar (IQ-TREE 2.4.0, LG+G, MAFFT L-INS-i alignment, ClipKIT trimming; rooting on characterised reference clades). The alignment and tree files accompany this figure in the Supplementary Data.
